# Challenges of II‐VI and III‐V Blue Quantum Dot Light‐Emitting Diodes

**DOI:** 10.1002/adma.202512379

**Published:** 2025-09-22

**Authors:** Shaun Tan, Jonah R. Horowitz, Oliver J. Tye, Moungi G. Bawendi

**Affiliations:** ^1^ Department of Chemistry Massachusetts Institute of Technology Cambridge MA 02139 USA

**Keywords:** blue quantum dots, colloidal quantum dots, operational stability, quantum dot light‐emitting diodes, quantum dot synthesis

## Abstract

Quantum dot light‐emitting diodes (QD‐LEDs) are electroluminescent devices where the emissive layer consists of inorganic colloidal quantum dots. Recent breakthroughs have enabled the development of bright and efficient blue‐emitting QD‐LEDs based on heavy metal‐free compositions. However, challenges remain that hinder their practical application in electroluminescent displays and lighting technologies. The primary obstacle is their limited operational lifetimes which remain significantly below practical requirement standards, especially in comparison to the red‐ and green‐emitting QD‐LEDs. Another important issue is the low color purity and broad spectral linewidths of heavy metal‐free blue quantum dot compositions. Additional problems include transient electroluminescent behaviors such as fluorescence intermittency and positive aging effects. This review examines the current understanding of the physical mechanisms underlying these challenges faced by blue QD‐LEDs. Often, contradictory explanations are proposed to account for the same phenomenon. Here, potential interpretations are suggested that may help reconcile the conflicting reports. Recent advances are further examined that have contributed to the development of state‐of‐the‐art blue QD‐LEDs.

## Graphical abstract

Blue‐emitting electroluminescent quantum dot light‐emitting diodes (QD‐LEDs) face critical challenges that limit their practical application, including short operational lifetimes, low color purity, broad spectral linewidths, and unpredictable transient effects like blinking and positive aging. This review explores the underlying mechanisms behind these challenges, discussing key knowledge gaps that require further investigations, and highlighting recent developments of state‐of‐the‐art blue QD‐LEDs.

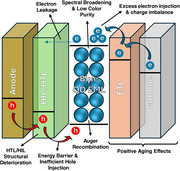

## 1 Introduction

Quantum dots (QDs) are semiconductor nanocrystals typically 2 to 10 nm in diameter that exhibit size‐dependent optoelectronic properties due to quantum confinement.^[^ ^1^ ^]^ QD color converters (QD‐CC) which are photoluminescent films that down‐convert blue photons from a backlight into red and green wavelengths have achieved commercial success and are widely found in modern display technologies to enable enhanced color saturation.^[^ ^2^, ^3^ ^]^ On the other hand, electroluminescent quantum dot light‐emitting diodes (QD‐LEDs) are still in the research and development phase and not commercialized, but QD‐LEDs represent the next generation technology that will harness the full potential of QDs by using them as full‐color self‐emissive materials and eliminating the need for a backlight. Compared to QD‐CC and competing organic light‐emitting diodes (OLEDs), QD‐LED technology promises a wider color gamut, superior color fidelity, more efficient energy consumption, and lower manufacturing costs,^[^ ^4^, ^5^ ^]^ positioning QD‐LEDs as strong contenders for future display and lighting applications.

Important developments in material synthesis and device engineering over the years^[^ ^6^, ^7^, ^8^, ^9^, ^10^, ^11^, ^12^, ^13^, ^14^ ^]^ have culminated in QD‐LEDs of all three primary colors (red, green, blue) with external quantum efficiencies (EQEs) exceeding 20%, high brightness, and heavy metal‐free compositions.^[^ ^15^, ^16^, ^17^, ^18^, ^19^, ^20^, ^21^, ^22^, ^23^, ^24^ ^]^ For context, the maximum theoretical QD‐LED EQE is estimated to be roughly 20–30%.^[^ ^25^, ^26^, ^27^, ^28^ ^]^ For blue‐emitting QD‐LEDs, several critical challenges remain that prevent their practical application. The most pressing issue is their limited operational stability, with best‐reported T_50_ operational lifetimes (time for luminance to decrease to 50% of its initial value) on the order of 10^4^ h at an initial brightness of 100 cd m^−2^, even for research‐scale spin‐coated devices.^[^ ^15^, ^18^, ^19^, ^22^, ^29^ ^]^ For comparison, the T_50_ lifetimes of red and green QD‐LEDs are orders of magnitude higher and exceed 10^8^ and 10^6^ h, respectively, at the same initial brightness.^[^ ^16^, ^17^, ^18^, ^20^, ^24^ ^]^ As a benchmark, blue‐emitting OLEDs used in commercial pixelated displays demonstrate T_50_ lifetimes of ≈10^6^ h under the same brightness conditions.^[^ ^30^ ^]^ While numerous degradation pathways have been proposed to explain the short lifetimes of blue QD‐LEDs, there remains no consensus understanding regarding their instability, which slows down progress in the research field.

Another significant challenge is the low color purity of blue QD‐LEDs,^[^ ^15^, ^31^ ^]^ which limits their contribution to the overall color gamut and compromises the color rendering quality for high‐definition display applications. In addition, many reported blue QD‐LEDs in the literature exhibit electroluminescent (EL) emission outside the desired pure blue wavelength range of 460 – 475 nm,^[^ ^15^, ^29^, ^32^ ^]^ which further leads to reduced visual sharpness and less vibrant color saturation. Besides operational lifetime and color purity, blue QD‐LEDs also suffer from transient effects that affect their EL behavior, such as fluorescence intermittency^[^ ^33^ ^]^ and positive aging effects,^[^ ^34^ ^]^ which occur across variable timescales and introduce unpredictability and inconsistency to the operation of blue QD‐LEDs. While red and green QD‐LEDs may undergo similar phenomena, these effects can be frequently more pronounced in blue QD‐LEDs as will be discussed.

**Figure 1:**
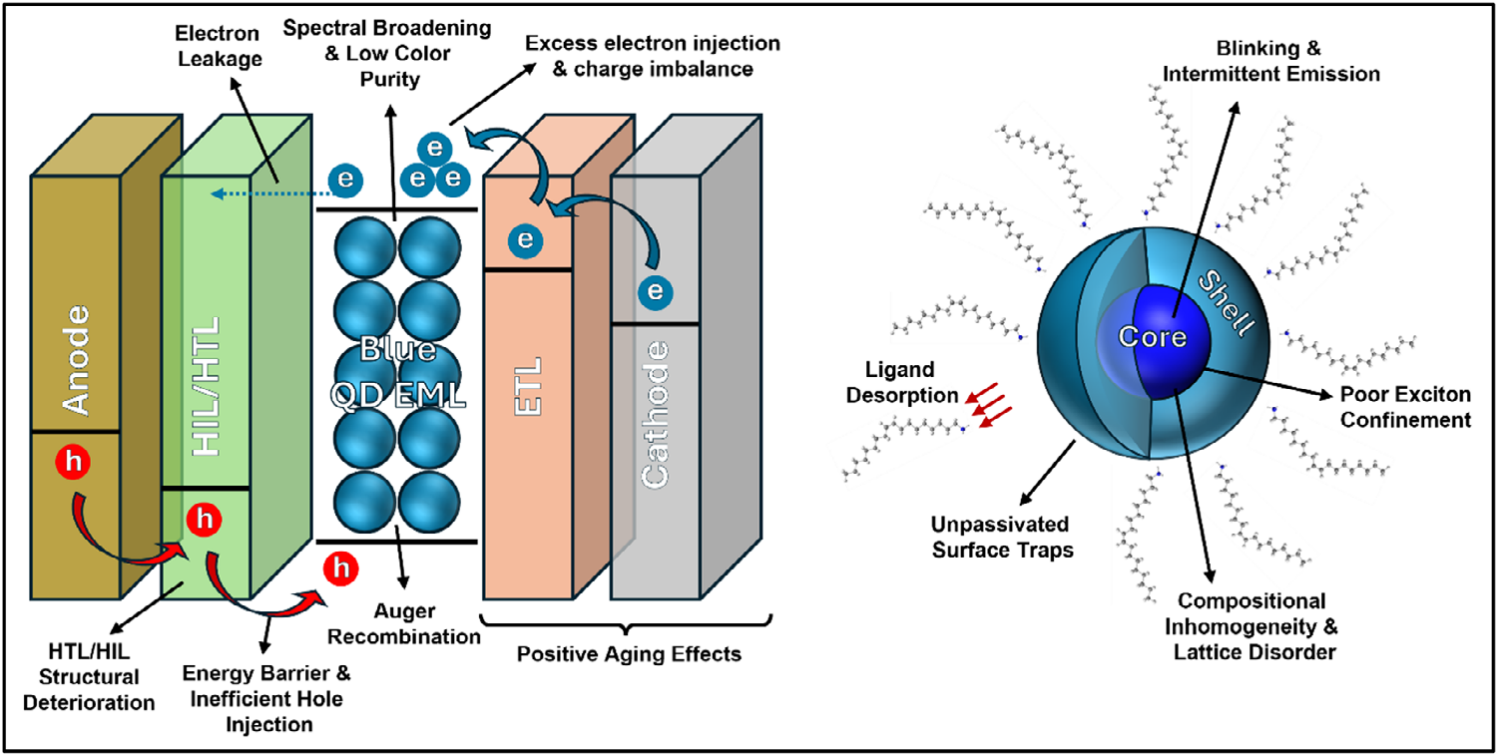
Schematic illustrating the challenges of blue quantum dot light‐emitting diodes.

In this review, we discuss the key challenges faced by blue QD‐LEDs (**Figure** **1**). Particular attention is given to discussing the distinct behaviors of blue QD‐LEDs compared to their red and green counterparts, as well as on the emerging insights from advanced mechanistic studies. Many proposed mechanistic explanations in the literature apply only to a particular blue QD composition or device structure, rather than being universally applicable. To further complicate matters, contradictory explanations can often be found in the literature to account for the same effect. We highlight key knowledge gaps and outstanding questions that require further investigations. We further evaluate recent breakthroughs in QD heterostructure design, material synthesis, charge transport layers, and device engineering, all of which have contributed to the development of state‐of‐the‐art blue‐emitting QD‐LEDs. Finally, we conclude by outlining promising general directions for future research.

## 2 Summary of Material Candidates for Blue QD Emitters

**Figure 2:**
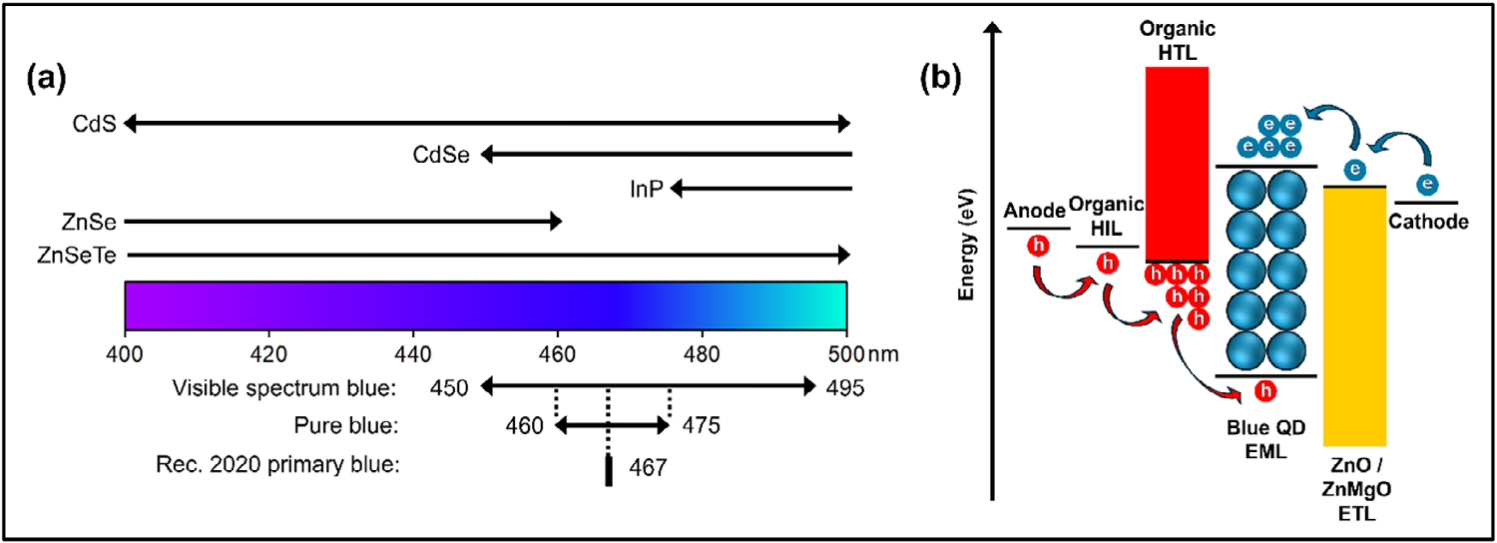
a) Schematic of the material candidates for blue‐emitting II‐VI and III‐V QD emitters. b) Schematic illustrating the general device structure and band alignment of the device layers.

**Figure** **2**a summarizes the candidate II‐VI and III‐V colloidal QD materials with blue EL emissions. Blue light is typically defined by the 450 – 495 nm wavelength range. Pure blue corresponds to a smaller subset within the range of 460 – 475nm. Shorter wavelengths (higher energy) are termed deep blue or violet, while longer wavelengths (lower energy) are labelled as sky blue or azure. The Rec. 2020 standard for Ultra High Definition (UHD) television is more specific and defines a precise blue primary color at 467 nm, corresponding to CIE (International Commission on Illumination) chromaticity coordinates x = 0.131, y = 0.046. For the discussions henceforth, unless specifically stated, “blue” refers to emissions in the entire wavelength range between 450 and 495 nm. Color purity as quantified by the EL emission spectrum linewidth (full width at half maximum, FWHM) is another important performance metric for QD‐LEDs. While there is no industry standard, the ideally narrow linewidth is typically considered to be <20 nm.

The most commonly reported blue QD‐LEDs are predominantly Cd‐based, with EQEs exceeding 25% and T_50_ operational lifetimes at 100 cd m^−2^ on the order of 10^4^ h for CdZnS and CdZnSe QDs.^[^ ^18^, ^22^, ^29^ ^]^ However, the use of Cd is heavily restricted by environmental regulations, particularly the European Union's Restriction of Hazardous Substances (RoHS) directive, which imposes strict limits on heavy metal content in consumer electronics. Specifically for Cd, the maximum permitted concentration is 0.01% (100 ppm) by weight.

Light‐emitting diodes based on lead halide perovskite materials^[^ ^35^, ^36^ ^]^ have also received attention for their high EL efficiency and narrow emission linewidths for all three primary colors. However, blue‐emitting perovskite LEDs generally exhibit shorter operational lifetimes than II‐VI and III‐V QD‐LEDs,^[^ ^37^, ^38^, ^39^ ^]^ and will also be hindered in practical application due to the presence of lead, another RoHS limited material. Therefore, this review primarily focuses on the II‐VI and III‐V quantum dot materials.

Heavy metal‐free III‐V InP QDs have seen great success as red and green emitters for high performance QD‐LEDs.^[^ ^16^, ^24^ ^]^ However, due to their small bulk bandgap (≈1.3 eV), to achieve blue emission the core diameters of InP QDs must be reduced to ≈1.5 – 2 nm to induce strong quantum confinement. Despite extensive research efforts, the EL efficiency remains poor for blue‐emitting InP QD‐LEDs due to synthetic challenges associated with their small cores that lead to non‐uniform size distribution, high surface defect densities, and lattice strain.^[^ ^40^, ^41^ ^]^ Alloyed InGaP QDs can achieve blue emission at relatively larger core sizes than pure InP.^[^ ^42^, ^43^ ^]^ However, their PLQY and color purity remain suboptimal and require further development.

Binary II‐VI ZnSe QDs are also heavy metal‐free, but their large bandgap (bulk bandgap: ≈2.7 eV) results in violet emission (<430 nm) that is poorly suited for practical applications. Enlarging the core size of ZnSe QDs can shift the emission wavelength into the blue regime. However, it is generally challenging to grow larger QD cores with isotropic shapes and uniform shelling,^[^ ^44^, ^45^ ^]^ and further synthetic developments are required in this aspect. On the other hand, alloying of Te into ZnSe can redshift the emission from 418 nm (pure ZnSe) to 496 nm (10% Te/Se doping content).^[^ ^31^ ^]^ Therefore, the emission wavelength and EL characteristics of ternary ZnSeTe QDs can be broadly tuned by varying both the core size and Te content. Since the first demonstration of a ZnSeTe‐based blue QD‐LED that surpassed 20% EQE,^[^ ^15^ ^]^ ZnSeTe QDs have received substantial attention as promising Cd‐free blue emitters, and recent developments have brought their performance and operational stability closer to those of leading Cd‐containing^[^ ^18^, ^22^, ^29^ ^]^ compositions. In addition to stability challenges, ZnSeTe QDs suffer from color purity issues such as broad spectral linewidths and asymmetric emission profiles, which will be discussed in detail below.

Figure 2b illustrates a typical QD‐LED device structure. **Table** **1** also lists the performance parameters of recent blue devices. The vast majority of QD‐LEDs in recent years typically construct a hybrid organic‐inorganic architecture, composed of an anode, an organic hole injection layer (HIL) especially poly(3,4‐ethylenedioxythiophene) polystyrene sulfonate (PEDOT:PSS), an organic hole transport layer (HTL) such as poly[(9,9‐dioctylfluorenyl‐2,7‐diyl)‐*alt*‐(4,4′‐(*N*‐(4‐butylphenyl))] (TFB), an emissive layer consisting of QD emitters, an inorganic electron transport layer (ETL) based on ZnO nanoparticles (inclusive of ZnMgO), and a metal cathode. Electrons and holes are injected through the electrodes and transport layers into the emissive layer to generate excitons, which subsequently undergo radiative recombination to emit photons. Interlayers can be inserted at any of the device interfaces for passivation or to regulate charge injection. Balancing the charge injection is essential for high performance and operationally stable QD‐LEDs. Generally for blue QD‐LEDs, hole injection is hindered by a large energy barrier at the QD/HTL interface caused by the deep valance band maximum (VBM) of the emissive layer. On the other hand, electron injection is favored because of the small conduction band minimum (CBM) gap at the QD/ETL interface and high electron mobility of ZnO and ZnMgO ETLs. The net result is an excess electron injection and charge imbalance in the emissive layer (Figure 2b), which will be a recurring theme discussed in detail in subsequent sections.

**Table 1:** Performance parameters of recent blue‐emitting QD‐LEDs.

	QD structure	Device structure	EL wavelength [nm]	EL FWHM [nm]	EQE [%]	T_50_ at 100 cd m^−2^ [h]	Refs.
ZnSeTe	ZnSeTe(7%)/ZnSe/ZnS	ITO/PEDOT:PFI/PVK/ QD/ZnMgO/Al	465	–	4	–	[46]
	ZnSeTe/ZnSe/ZnS	ITO/PEDOT/TFB/ QD/ZnMgO/Al	460	35	20.2	15850	[15]
	ZnSeTeS/ZnSe/ZnS	ITO/PEDOT/PF8Cz/ QD/ZnMgO/Al	460	17	24.7	29600	[19]
	ZnSeTeS/ZnSe/ZnS	ITO/PEDOT/PF8Cz/ QD/ZnMgO/Al	468	25	24.6	17500	[19]
	ZnSe	ITO/PEDOT/PVK/ QD/ZnMgO/Al	445	<12	12.2	237	[47]
Cd‐based	ZnCdSe/ZnSeS/ZnCdS/ZnS	ITO/PEDOT/TFB/ QD/ZnMgO/Al	478	19	19.7	50206	[22]
	ZnCdSe/ZnSeS/ZnCdS/ZnS	ITO/PEDOT/TFB/ QD/ZnMgO/Al	482	22	20.4	80377	[22]
	ZnCdSe/ZnSeS/ZnCdS/ZnS	ITO/PEDOT/TFB/ QD/ZnMgO/Al	467	20	20.1	–	[22]
	ZnCdSe/ZnSeS/ZnCdS/ZnS	ITO/PEDOT/TFB/ QD/ZnMgO/Al	471	20	20.8	–	[22]
	CdZnSe/ZnS	ITO/PEDOT/PF8Cz/ QD/ZnMgO/Al	479	23	21.9	24000	[18]
	ZnCdS/Cd*_x_* Zn_1−_ *_x_* S/ZnS	ITO/PEDOT/PVK/ QD/ZnO/Al	445	19	18.0	47.4	[48]
	Cd_1−x_Zn_x_S	ITO/PEDOT/TFB/ QD/ZnO/Al	467	–	12.5	–	[49]
	CdSe/ZnSe	ITO/PEDOT/TFB/ QD/ZnO/Al	477	–	8.05	7000	[50]
	CdSe/ZnSe/ZnS	ITO/PEDOT/PF8Cz/ QD/EDTAK ZnO/Al	468	–	16.31	1685	[51]
	ZnCdS/ZnS	ITO/PEDOT/CBP‐V:T5DP‐2,7/ QD/ZnO:PVP/Al	461	20	18.59	502	[52]
	CdSe/ZnSe/ZnS	ITO/PEDOT/PVK:C_8_‐SS/QD/ZnMgO/Al	464	–	19.02	1183	[53]
	CdSe/ZnS	ITO/PEDOT/PVK/ QD/ZnO/Al	468	20	19.8	47.4	[54]
	ZnCdS/ZnS	ITO/PEDOT/TFB/ QD/ZnO/Al	445	25	15.6	–	[55]
	ZnCdSe/ZnS//ZnS	ITO/PEDOT/TFB/ QD/PMMA/ZnO/Al	472	34	16.2	355	[56]
	g‐CdZnSeS/ZnS	ITO/PEDOT/PVK/ QD/ZnMgO/Al	479	–	24	27000	[57]
	CdZnS/ZnS	ITO/PEDOT/TFB/PBO/ QD/ZnMgO/Al	458	26	23	41000	[29]
InP	InP/ZnS	ITO/ZnMgO/QD/ CBP/MoO_3_/Al	488	45	–	–	[58]
	InP/GaP/ZnS//ZnS	ITO/PEDOT/TFB/ QD/ZnO/Al	488	50	1.01	2	[16]
	InGaP	ITO/PEDOT/PVK/ QD/ZnMgO/Al	469	–	2.5	–	[42]

## 3 Challenges and Dynamic Electroluminescence Behaviors of Blue QD‐LEDs

### 3.1 Operational Instability

**Figure 3:**
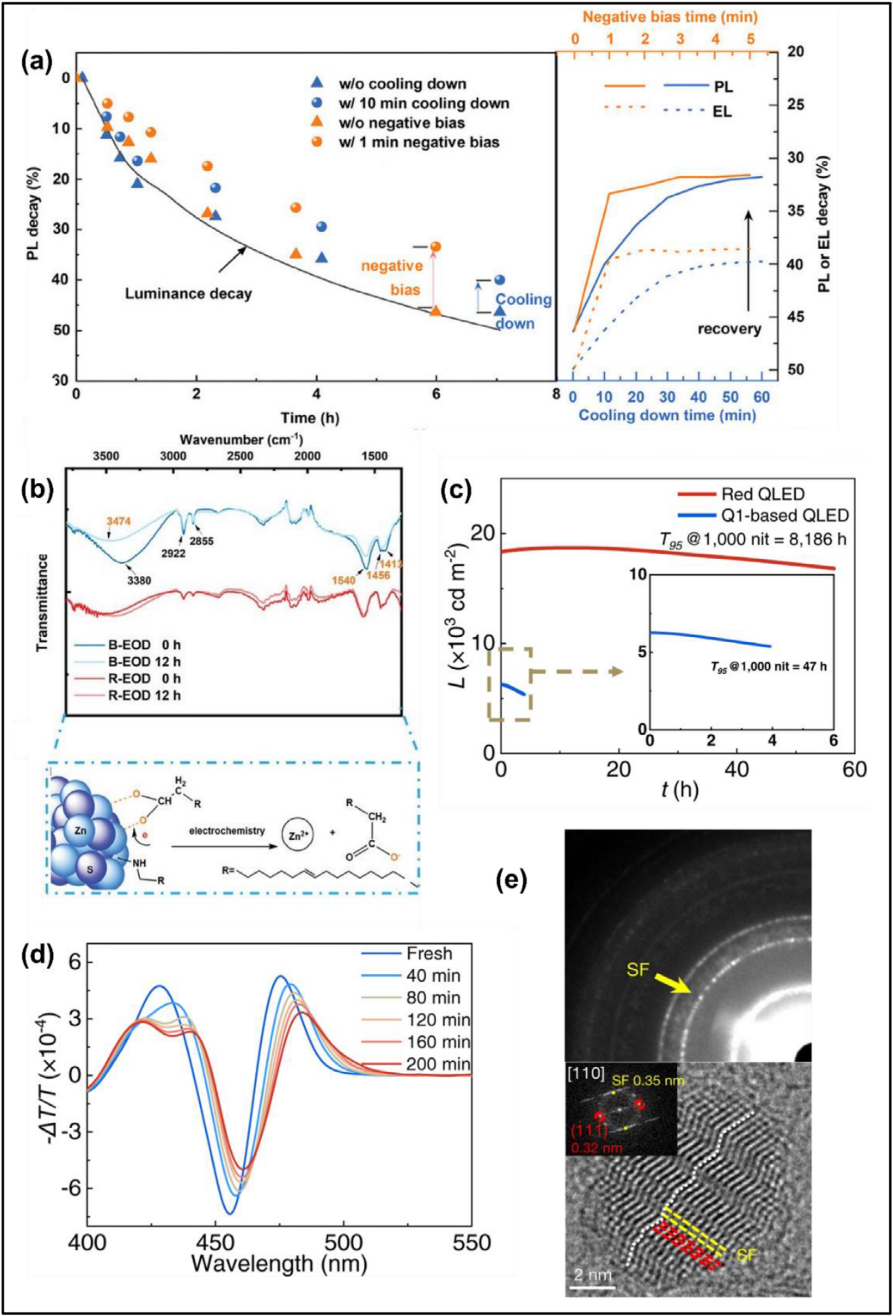
a) Evolution of the PL and EL of blue‐emitting CdSe/ZnSe/ZnS QD‐LEDs at different aging times. The right side shows the PL and EL recovery by powering off and cooling the QD‐LED (in blue) or by applying a negative voltage (in orange). Reproduced with permission.^[^ ^59^ ^]^ Copyright 2023, Wiley‐VCH. b) FTIR spectra of the blue and red electron‐only devices before and after aging at a constant current of 0.4 mA. Reproduced with permission.^[^ ^59^ ^]^ Copyright 2023, Wiley‐VCH. c) Operational lifetime comparison of red and blue QD‐LEDs at a constant current density of 150 and 50 mA cm^−2^, respectively, and d) evolution of the electroabsorption spectra of the blue QD‐LEDs at a constant current density of 100 mA cm^−2^. Reproduced with permission.^[^ ^22^ ^]^ Copyright 2023, Nature Publishing Group. e) Selected‐area diffraction pattern (left) and high‐resolution TEM image (right) of ZnSeTe/ZnSe/ZnS QDs showing the stacking faults (in yellow) compared to the normal zinc blende crystal structure (in red). Reproduced with permission.^[^ ^15^ ^]^ Copyright 2020, Nature Publishing Group.

The operational instability of blue QD‐LEDs is the most urgent challenge preventing their practical application. As discussed, the best‐reported T_50_ lifetimes at 100 cd m^−2^ for blue QD‐LEDs are on the order of 10^4^ h,^[^ ^15^, ^18^, ^19^, ^22^, ^29^ ^]^ which lag significantly behind those of red (10^8^ h) and green (10^6^ h) QD‐LEDs at the same initial brightness level.^[^ ^16^, ^17^, ^18^, ^20^, ^24^ ^]^ During QD‐LED degradation, a decreasing EL efficiency is often accompanied by an increasing operatingcd voltage. In other words, a higher driving voltage is needed to maintain a constant current density, implying that the current injection is being hindered and the device resistivity is increasing. The key goal is to identify which specific device layer (or combination of multiple layers) is responsible for the overall QD‐LED degradation. Barring any transient effects which will be described in the subsequent sections, some reports have shown that blue QDs could maintain relatively steady PL under photoexcitation.^[^ ^59^, ^60^, ^61^ ^]^ One specific analysis of ZnSeTe/ZnSe/ZnS blue QD‐LEDs showed that the PL intensity was still maintained at 85% even after the EL decreased to 50% of its initial value.^[^ ^15^ ^]^ Some additional reports have observed similar results across various blue QD compositions including ZnSeTe, CdSe, and CdZnSeS (**Figure** **3**a).^[^ ^59^, ^60^, ^61^ ^]^ These observations indicate that the intrinsic chemical and material degradation of blue QDs only partially contributes to device failure. Unlike photoexcitation where the exciton is photogenerated and mostly confined within the QD core, EL requires electron and hole injection through multiple transport layers. To ensure operationally stable QD‐LEDs, it is crucial that the transport layers, emissive layer, and all associated interfaces remain robust and functional during long‐term operation.

#### 3.1.1 Emissive Layer and Surface Ligands

Substantial progress has been made in the stability of colloidal QDs under excitation conditions since their first uniform and controlled synthesis.^[^ ^62^ ^]^ Due to a high surface area to volume ratio, the QD electronic structure is particularly susceptible to electronic trap states associated with an undercoordinated surface resulting in PLQY loss. Additionally, such surface sites are more likely to react with surrounding chemical species, such as oxygen, resulting in poor stability. Shelling with a high bandgap material^[^ ^63^ ^]^ and passivation with organic capping ligands has resulted in drastically enhanced QD performance compared to early work. However, dynamic behavior in the QD surface environment during device operation still presents challenges to the stability of the emissive layer in QD‐LEDs.

The accumulation of excess electrons in the QD emissive layer is frequently described as a key contributing factor to the instability of blue QD‐LEDs. In blue devices, electron accumulation may occur due to the large energy gap (≈1 eV) at the QD/HTL interface, hindering hole injection (Figure 2b). In turn, excess electrons may induce the detachment of ligands from the QD surface.^[^ ^64^ ^]^ Prototypical capping ligands used for blue QDs, such as oleic acid, 1‐dodecanethiol, and 1‐octanethiol, have been shown by some reports to be vulnerable to electrochemical redox reactions,^[^ ^59^, ^65^, ^66^ ^]^ leading to irreversible desorption from the QD surface. Higher operating temperatures further aggravate the ligand detachment process and accelerate device failure.^[^ ^23^, ^67^ ^]^ The loss of capping ligands may leave behind unpassivated surface traps that act as non‐radiative recombination centers and reduce the PLQY. The detachment of oleic acid ligands by electrons was experimentally observed through Fourier transform infrared spectroscopy (FTIR) studies on electron‐only devices based on blue CdSe/ZnSe/ZnS QDs by monitoring the change in the ‐COO‐ intensity between 1540 and 1413 cm^−1^ (Figure 3b).^[^ ^59^ ^]^ FTIR further observed that oleic acid detachment is much less severe for the red CdSe/ZnSe/ZnS QDs. The report suggested that the strongly‐confined Cd‐based blue QDs with small core sizes and high specific surface areas experienced a higher degree of electron‐ligand interactions due to their poor passivation and higher surface ligand density.^[^ ^59^ ^]^

Surface trap states act as non‐radiative recombination centers that quench excitons and reduce the EL efficiency over time. It was suggested that the poor exciton confinement and strong surface‐bulk coupling of blue Cd‐based QDs with small core diameters exacerbate the detrimental effects of surface localized charges.^[^ ^22^, ^57^ ^]^ As the surface‐to‐volume ratio is higher for small cores, the exciton dynamics can be more affected by surface states. This has been proposed to explain the short lifetimes of blue ZnCdSe/ZnCdSeS/ZnS devices with small cores in comparison to larger red QDs with lower surface‐to‐volume ratio and negligible surface‐bulk coupling (Figure 3c).^[^ ^22^ ^]^ Electroabsorption measurements of the blue QD‐LEDs show a time‐dependent redshift of the first excitonic peak (Figure 3d), which was interpreted as an increase in optically dark transitions associated with surface states.^[^ ^22^ ^]^ On a similar note, compositional inhomogeneity and lattice instability caused by Te incorporation have more recently been the focus of research efforts for ZnSeTe‐based QDs.^[^ ^19^, ^46^ ^]^ The large radius mismatch (Se: 1.98 Å, Te: 2.21 Å) and high reactivity of Te precursors during synthesis may cause phase segregation of Te‐rich clusters, and the QDs have been observed^[^ ^31^, ^33^ ^]^ to form severe stacking faults (Figure 3e) that act as non‐radiative recombination centers which lead to exciton quenching. While Te aggregation, stacking faults, and structural inhomogeneity may be important factors for ZnSeTe QDs, these are however not a universal explanation for the instability of blue QD‐LEDs, since compositions that do not contain Te (e.g., the Cd‐based blue QDs such as CdSe or CdZnSeS) suffer from short device operational lifetimes.

While blue‐emitting QDs with extremely small cores (diameter <2 nm) may suffer from more severe ligand detachment or poor exciton confinement, how their severity correlate with the blue QD core size remains poorly understood. Systematic studies that could explicitly demonstrate that such degradation mechanisms are exacerbated on blue QDs with smaller cores would be valuable. More broadly, alternative studies have observed ligand detachment for red and green QD‐LEDs,^[^ ^64^, ^65^ ^]^ yet their operational stabilities remain significantly longer than the blue devices. The question is then on how the ligand detachment processes differ between blue QDs versus red and green emitters. Ligand detachment is expected to decrease the PLQY, but linking back to the previous discussion on the PL and EL gap, it is necessary to understand how much ligand detachment contributes to the EL loss of blue‐emitting QD‐LEDs. Meanwhile, excess electron accumulation is also an issue in red and green QD‐LEDs, since typical ZnO‐based ETLs have higher electron mobility compared to the low hole mobility of common polymer or small molecule HTLs.^[^ ^13^, ^68^, ^69^ ^]^ As mentioned, the wider bandgap of blue QDs has been suggested to worsen inefficient hole injection. However, this explanation may be insufficient because while the cores of blue QDs have larger bandgaps, the shell structures and surface ligands, with their own intrinsic bandgaps and charge mobilities, may be similar to those used in red and green QDs. Some reports also show that the effective VBM of blue QDs measured experimentally (e.g., −6.0 eV for ZnSeTe)^[^ ^15^, ^19^ ^]^ is comparable to the values reported for red and green QDs.^[^ ^27^, ^70^, ^71^ ^]^ Given these contrasting explanations, more mechanistic studies are necessary to investigate the band alignments and differentiate the degradation processes between blue QD‐LEDs versus their red and green counterparts.

#### 3.1.2 Degradation of the Charge Transport and Injection Layers

**Figure 4:**
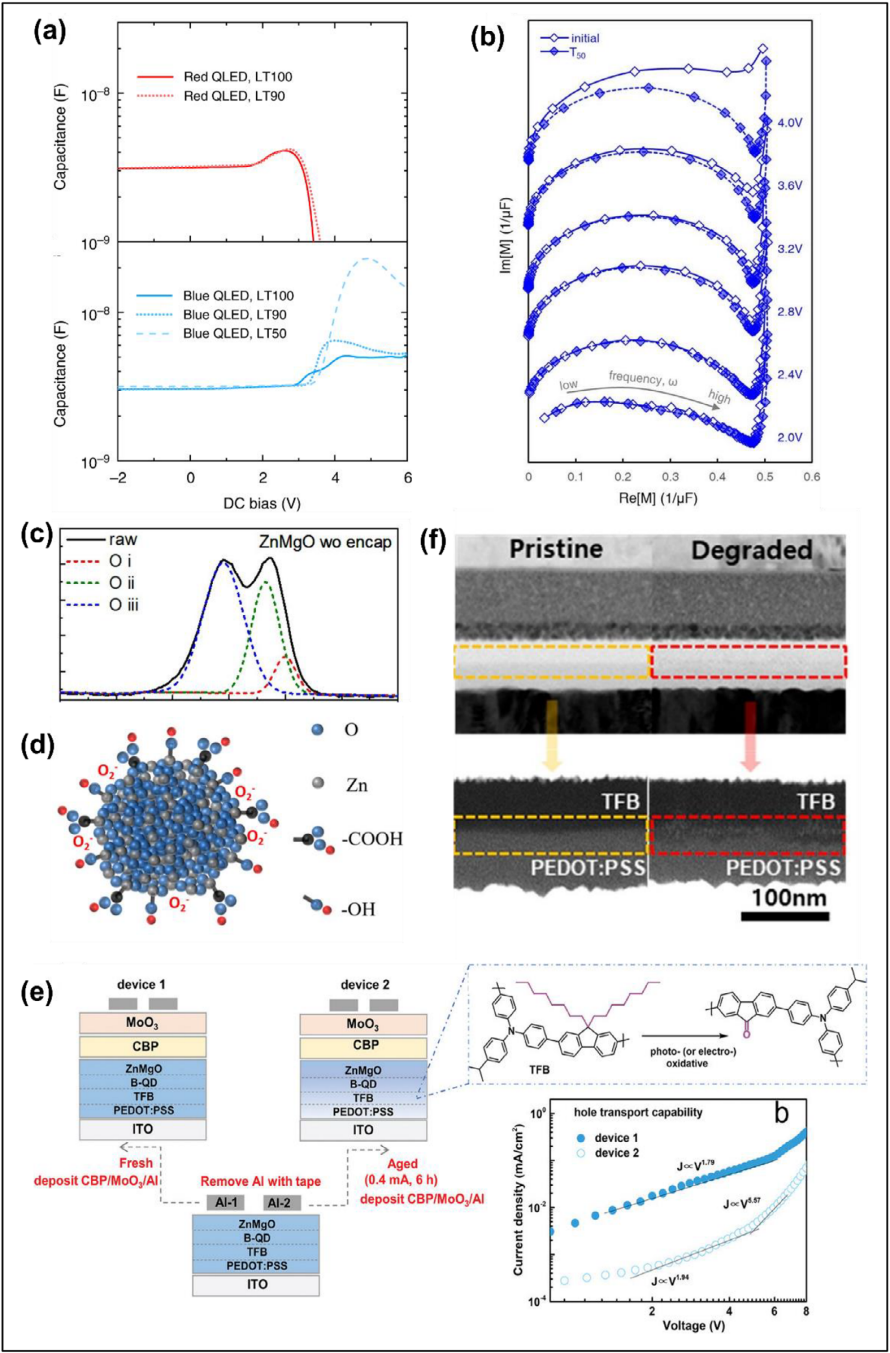
a) Capacitance‐voltage characteristics of the red and blue QD‐LEDs before and after lifetime testing. Reproduced with permission.^[^ ^49^ ^]^ Copyright 2019, Nature Publishing Group. b) Complex modulus spectra at different voltages for the pristine and aged ZnSeTe/ZnSe/ZnS blue QD‐LEDs. Reproduced with permission.^[^ ^15^ ^]^ Copyright 2020, Nature Publishing Group. c) XPS spectra, and d) schematic diagram of ZnMgO showing the large number of surface states. Reproduced with permission.^[^ ^73^ ^]^ Copyright 2023, American Chemical Society. e) Schematic of the “peel off” process on blue CdSe/ZnSe/ZnS QD‐LEDs to create single‐carrier hole‐only devices to study the degradation of TFB. Reproduced with permission.^[^ ^59^ ^]^ Copyright 2024, Wiley‐VCH. f) Cross‐sectional TEM images of the QD‐LEDs before and after constant current operation at 7 mA for 55 h. Reproduced with permission.^[^ ^80^ ^]^ Copyright 2021, American Chemical Society.

While the nature of QD degradation is still under debate, several experiments that monitor both the PL and EL intensity as a function of device operation have demonstrated that much larger intensity decays are observed in the EL compared to the PL as discussed in section 3.1, suggesting the deterioration of the functional layers surrounding the emissive layer. Some reports suggested that the ZnO ETL majorly contributes to the degradation of blue QD‐LEDs. For pure ZnO, a large type II heterojunction (Figure 2b) is formed at the blue QD/ZnO interface due to the wide bandgap of blue QDs and deep conduction band minimum (CBM) of ZnO. The relatively upshifted CBM of the blue QD and type II band alignment allows undesirable electron back‐transfer due to the built‐in electric field at the interface and space charge accumulation at the QD/ZnO interface and ZnO. The electron back‐transfer has been linked to the rise in operating voltage and degradation of CdZnS blue QD‐LEDs as inferred using capacitance‐voltage measurements (**Figure** **4**a).^[^ ^49^ ^]^ Systematic comparisons in the same study suggested that electron back‐transfer from red‐emitting CdZnSeS QDs to ZnO was prevented by their smaller bandgap which avoided the type II band alignment.^[^ ^49^ ^]^ More recent blue QD‐LEDs use ZnMgO as the ETL instead of pure ZnO. Alloyed ZnMgO has a larger bandgap and shallower CBM than ZnO (−3.4 eV for ZnO, −3.2 eV for Zn_0.9_Mg_0.1_O), which is expected to facilitate electron injection into the emissive layer and suppress electron back‐transfer. However, some studies of single‐carrier electron‐only devices with ZnMgO show that the voltage continuously decreased with time under electron injection, suggesting that ZnMgO is vulnerable to electron stress.^[^ ^72^ ^]^ Likewise, one report on the post‐mortem analyses of ZnSeTe/ZnSe/ZnS blue QD‐LEDs using impedance spectroscopy (Figure 4b) suggested that the increased resistance may have originated from the ETL.^[^ ^15^ ^]^ While the instability of ZnMgO is noted in several of these studies, the physical processes of ZnMgO degradation remain unclear. Meanwhile, both ZnO and ZnMgO have high intrinsic defect densities that may constitute charge traps and leakage channels.^[^ ^70^, ^73^, ^74^ ^]^ In particular, the abundant oxygen vacancy and zinc interstitial defects, and residual precursors such as acetate and hydroxyl groups leftover after synthesis (Figure 4c,d),^[^ ^73^ ^]^ may play important roles in ZnO and ZnMgO degradation and long‐term blue QD‐LED operational stability.

In addition, the instability of blue QD‐LEDs has also been linked with changes associated with the organic HTL. The large energy barrier at the QD/HTL interface (≈1 eV) (Figure 2b) leads to inefficient hole injection into the emissive layer and consequent hole accumulation in the HTL. Some reports have linked the short lifetimes of blue QD‐LEDs with HTL oxidation and damage by accumulated holes,^[^ ^29^ ^]^ or trap formation and structural deterioration that cause exciton quenching.^[^ ^75^, ^76^ ^]^ One study on blue QD‐LEDs based on CdZnS/ZnS suggested that hole accumulation at the QD/HTL interface caused by the inefficient hole injection promoted HTL oxidation, which in turn limited the device operational lifetime.^[^ ^29^ ^]^ Conflictingly, other reports proposed that hole accumulation and HTL oxidation are not major contributors to the operational instability of blue devices.^[^ ^49^, ^77^ ^]^ A post‐mortem analyses of degraded blue CdZnS QD‐LEDs after lifetime testing detected oxidized TFB species using electroabsorption and capacitance‐voltage techniques.^[^ ^49^ ^]^ Despite the oxidized TFB, the study observed that the operating voltage rise across TFB was minor, which was used as evidence to suggest that the HTL may not be a major degradation factor for blue QD‐LEDs.^[^ ^49^ ^]^ Some recent findings^[^ ^78^, ^79^ ^]^ suggested that optimizing the HTL/electrode energy alignment, even at the expense of a worst QH/HTL offset, may still contribute to improving operational stability. This was attributed to the significant role of Fermi level pinning to impact the hole injection barrier. Meanwhile, optimizing the HTL/electrode energy alignment promoted *p*‐doping and hole conductivity to effectively achieve better charge balance.^[^ ^78^ ^]^ Such insights may contribute to help reconcile the conflicting observations regarding the role of the HTL, and also suggest that a holistic approach involving the HTL, HIL, and electrode may be necessary to improve hole injection for blue QD‐LEDs.

While the consequences of hole accumulation are still being debated, electron leakage from the emissive layer is generally accepted as a major cause of instability for the organic HTLs and HILs used in QD‐LEDs including the red and green devices.^[^ ^18^, ^77^, ^81^ ^]^ Despite the energy barrier against electron transfer at the QD/HTL interface, electrons may tunnel into the deep tail states and broadened LUMO levels induced by traps and structural disorders of the HTL.^[^ ^18^, ^82^, ^83^ ^]^ Electron leakage is commonly observed by PL emission from the HTL.^[^ ^18^, ^82^, ^83^ ^]^ Electron leakage can lead to various problems that cause HTL and HIL degradation, including electrochemical reduction reactions,^[^ ^77^, ^84^ ^]^ structural deformation,^[^ ^80^ ^]^ and pinhole formation.^[^ ^85^ ^]^ These effects have been universally observed for various organic HTLs typically used in QD‐LEDs, such as TFB, 4,4‐*N*,*N*‐dicarbazole‐biphenyl (CBP), and poly‐*N*‐vinylcarbazole (PVK).^[^ ^77^, ^81^, ^84^, ^85^, ^86^ ^]^ Specifically, organic polyfluorenes (inclusive of TFB) are highly vulnerable to electrochemical reactions induced by leakage electrons,^[^ ^87^ ^]^ which irreversibly decreases their hole mobility and conductivity. Electron leakage and exciton recombination at the QD/HTL interface rather than being confined within the QD emissive layer can also cause structural deterioration of the HTL.^[^ ^59^ ^]^ By conducting “peel off” and single‐carrier hole‐only device studies of the aged QD‐LEDs (Figure 4e), the degradation was attributed to TFB decomposition and reduction of its hole transport capability.^[^ ^59^ ^]^ For the commonly used PEDOT:PSS HIL, leakage electrons was observed to form voids and caused destruction of the HTL/PEDOT:PSS interface, which was detected using cross‐sectional TEM of red InP QD‐LEDs (Figure 4f).^[^ ^80^ ^]^ Overall, as the HTL and HIL become more resistive due to damage by leakage electrons, increased Joule heating aggravates the degradation of the organic materials with poor thermal stability.^[^ ^85^ ^]^ Furthermore, reduction of the HTL and HIL hole mobility then worsens charge imbalance and exacerbates inefficient hole injection into the emissive layer. Altogether, these degradation pathways associated with the HTL and HIL cascade together to accelerate QD‐LED failure. Ultimately, degradation of the HTL, HIL, and QD/HTL/HIL interfaces are thought to be the primary causes of device failure for red and green QD‐LEDs in the long‐term. Despite this, they remain significantly more stable than blue QD‐LEDs, which may suggest that HTL and HIL degradation may not be the intrinsic culprits responsible for the short operational lifetimes of blue devices, but more studies are required to explore this. Nevertheless, HTL and HIL development are still crucial, as will be discussed in section 4.

### 3.2 Rapid Initial Decay in Luminescence Intensity and Intermittent Emission

QD‐LEDs frequently exhibit transient behaviors during the early stages of long‐term operation. In some cases, a noticeable drop in luminescence intensity occurs within the first few hours of operation before the long‐term degradation trends set in. Such early‐stage transients present challenges for practical applications, where reproducible trends are necessary to predict and control the QD‐LED EL characteristics such as color rendering.

The initial intensity drop is largely attributed to charge accumulation in the QD emissive layer, which leads to rapid nonradiative Auger‐Meitner recombination, a mechanism closely related to fluorescence intermittency or “blinking” events observed in single QDs.^[^ ^88^ ^]^ Although the exact photophysical nature of blinking in QDs is still debated,^[^ ^89^ ^]^ the widely accepted model is built upon the premise that charged QDs are weakly emissive or non‐emissive (OFF) due to enhanced probability of non‐radiative Auger‐Meitner recombination, while neutral QDs recombine radiatively (ON).^[^ ^90^ ^]^ Changes in the QD charge state result is switching between these ON and OFF states. The duration of ON and OFF events are typically observed on the orders of seconds and minutes and are well‐described by truncated power laws, with the ON power law usually truncating at shorter times than the OFF.^[^ ^91^ ^]^ When taking PL intensity across a statistical average of many blinking QDs, this results in a photodarkening effect known as statistical aging.^[^ ^92^, ^93^ ^]^ In other words, since each QD in the ensemble has a probability of entering a long OFF state, a growing number of QDs will enter this state during the experimental time, leading to a photodarkening of the overall ensemble. Since this process is purely a charging effect and not caused by chemical degradation of the QDs, the process can be reversed when all QDs are allowed sufficient time to return to their ON state.^[^ ^92^ ^]^ The reversible nature of statistical aging provides a valuable method of distinguishing between non‐destructive charging behavior and true irreversible QD degradation that constitute the long‐term instability of QD‐LEDs.

The formation of charged QDs within QD‐LED devices has been observed by multiple reports.^[^ ^86^, ^94^, ^95^ ^]^ An early study^[^ ^94^ ^]^ showed the accumulation of excess electrons in a red CdSe/CdS QD‐LED through measurements of the time‐resolved PL lifetimes which closely matched that of the negative trion. Under reverse bias, the lifetime became longer, suggesting a decreased number of charged QDs.^[^ ^96^ ^]^ This behavior was tied to the QD‐LED initial intensity drop by a separate previous study.^[^ ^86^ ^]^ Here, the experiment measured the TRPL lifetime of CdSe/CdS QDs as the PLQY decreased over the course of 30 min. During the initial rapid luminance decay, the lifetime showed a short component with a time constant consistent with that of the negative trion. However, after applying a reverse bias, the PLQY returned to 93% of its original value and the short lifetime component disappeared, largely reversing the aging process and supporting a model of luminance decay driven by charge accumulation in the QD emissive layer. The generation of negatively charged QDs has also been observed in single‐QD devices^[^ ^95^ ^]^ and similar behavior has been reported in red InP/ZnSe/ZnS QD‐LEDs.^[^ ^97^ ^]^ Here, both complete QD‐LEDs and single‐carrier devices (hole‐only and electron‐only) were run for an hour with reverse bias applied every 15 min to discharge the devices. While the complete QD‐LED and hole‐only devices showed a slight recovery of luminance intensity after the discharging periods, the electron‐only device showed nearly complete (>90%) restoration of intensity, suggesting an excess of electrons caused reversible charging behavior, while an excess of holes induces permanent QD‐LED degradation. To distinguish between reversible charging behavior and irreversible QD damage in blue Cd‐based QD‐LEDs,^[^ ^22^ ^]^ a study combined measurements of EL and PL intensity decay with periodic pulses of reverse bias. By comparing the PL intensity of the aged device after a pulse of reverse bias to the initial intensity, the degradation of the QDs themselves could be distinguished from the charging processes. Interestingly, the study suggests that 100% PL could be recovered with reverse bias, or turning off and resting the device, only if the QD‐LED was operated for short periods of time (2 min).^[^ ^22^ ^]^ Meanwhile, longer operation periods resulted in some irreversible PL loss, despite stable EL and PL during long‐term operation.^[^ ^22^ ^]^ Such behavior suggests the occurrence of irreversible chemical dynamics when charges are accumulated for longer periods. In addition, the initial luminance drop may also be caused by early device operation effects, primarily due to Joule heating, which can be further intensified by thermal energy released through Auger–Meitner recombination processes. A study using temperature‐dependent PL attributed an observed luminance quenching to two processes: irreversible degradation from thermally‐induced structural changes and reversible quenching caused by thermally‐activated carrier trapping or creation of trap states.^[^ ^98^ ^]^ In a QD‐LED architecture, the report observed a small portion of the observed initial luminance drop could be recovered with cooling, rather than reverse‐bias alone, supporting a thermally‐activated mechanism.^[^ ^86^ ^]^ In summary, these examples illustrate the value of combining EL and PL measurements to disentangle the underlying causes of QD‐LED luminance loss and to assess whether the performance drop reflects irreversible degradation or reversible transient effects.

The initial luminance drop in QD‐LEDs is a common feature attributed to charge‐accumulation and heat generation, distinguishable from longer term degradation by its reversible nature. Although it is often not given much attention, studies comparing red and green devices to blue QD‐LEDs may reveal differing susceptibilities of blue QDs to charge accumulation and heat. For example,^[^ ^22^ ^]^ a systematic comparison showed that a red ZnCdSe/ZnCdSeS/ZnS reference device undergoes a smaller initial luminance drop compared to their blue QD‐LEDs. More work is needed to understand whether these differences can be tied to longer term degradation mechanisms.

### 3.3 Positive Aging Effects

Positive aging represents an initial increase in device brightness and EL efficiency with time. This behavior is opposite to the rapid initial luminescence decay discussed previously in section 3.3. While such performance improvements may appear advantageous, the magnitude and duration of positive aging vary, seemingly at random across different QD‐LEDs and device batches, making it difficult to achieve consistent and reliable EL performance. Predictability and reproducibility are essential for a commercial product, and thus understanding the mechanisms behind positive aging and controlling its occurrence are important. It is necessary to differentiate two types of positive aging. During initial device operation, positive aging may occur over shorter timescales of less than a few hours. In contrast, another form of slow positive aging can happen during device shelf storage and may be prolonged over a period of days and even weeks. The latter form of positive aging may be accelerated by device operation and convolved with the first type of positive aging. There is still active debate in the community regarding the underlying physical causes for both types of positive aging.

**Figure 5:**
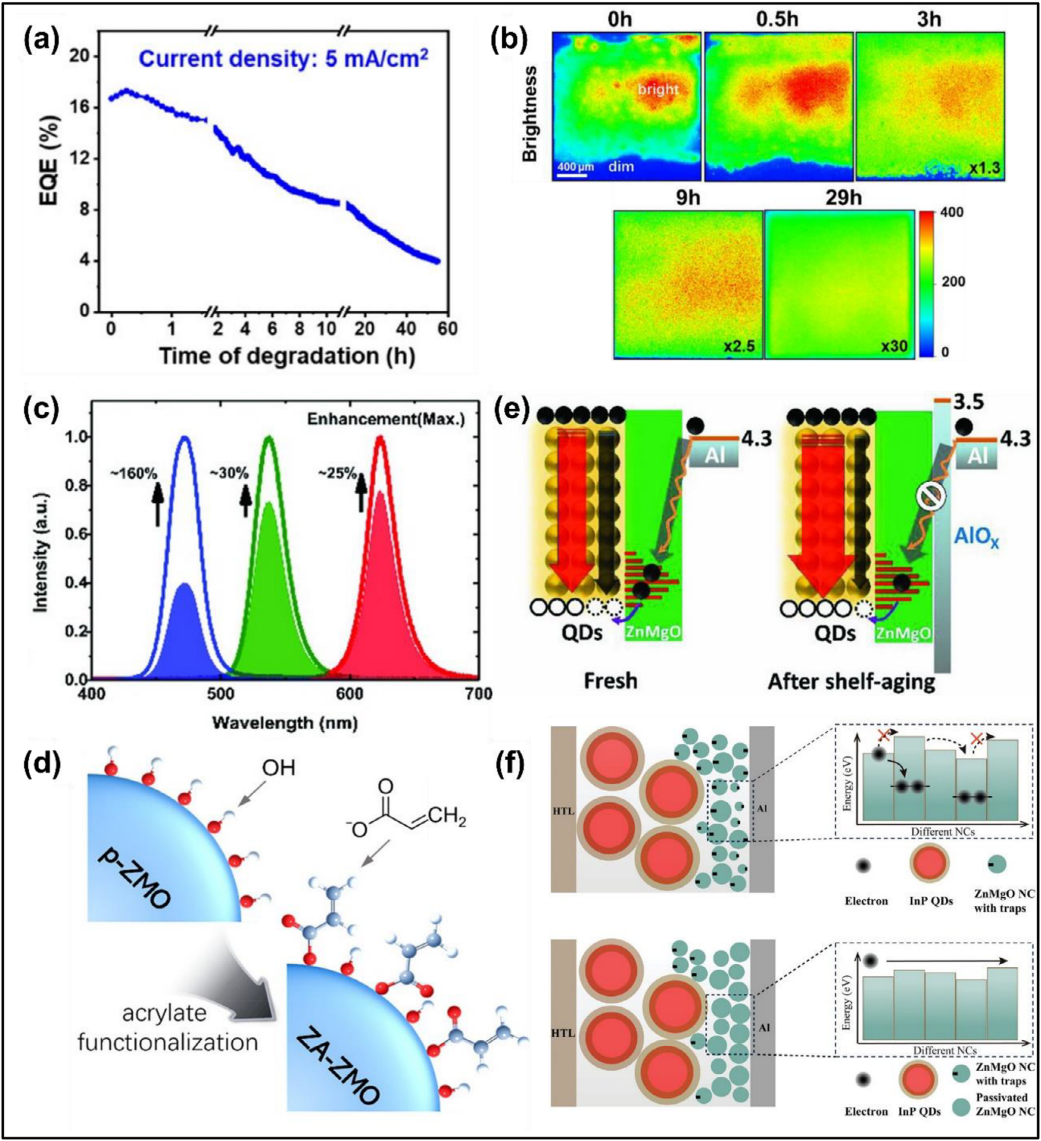
a) EQE change of the QD‐LED with time under at a constant current density of 5 mA cm^−2^, and b) time‐resolved EL mapping of the QD‐LED brightness with time. Reproduced with permission.^[^ ^99^ ^]^ Copyright 2025, Wiley‐VCH. c) Change in EL of the red‐, green, and blue‐emitting QD‐LEDs after positive aging. Reproduced with permission.^[^ ^34^ ^]^ Copyright 2018, Wiley‐VCH. d) Schematic of the surface of ZnMgO after acrylate functionalization. Reproduced with permission.^[^ ^74^ ^]^ Copyright 2022, American Chemical Society. e) Band alignment showing the formation of AlO_x_ during positive aging and suppressed exciton quenching at the Al/ZnMgO interface. Reproduced with permission.^[^ ^34^ ^]^ Copyright 2018, Wiley‐VCH. f) Schematic illustrating the size ripening process of ZnMgO nanocrystals, improved uniformity, and more homogenous energy profile after the positive aging effect. Reproduced with permission.^[^ ^100^ ^]^ Copyright 2025, Wiley‐VCH.

Some reports explained the positive aging during initial device operation with a model of charge neutrality restoration.^[^ ^11^, ^13^ ^]^ In one example, QD emitters began in a positively‐charged dark state due to electron transfer to ZnO, and during operation electrons were injected back into the emissive layer to restore charge balance and increase the EL efficiency over a period of ≈1 h.^[^ ^11^ ^]^ More recent developments attribute the positive aging during operation to a sequential exciton generation process.^[^ ^99^, ^101^ ^]^ Time‐resolved EL spectroscopy was used to study the positive aging effect of blue CdSe/CdZnSeS/ZnS QD‐LEDs, where the EQE continuously increased during initial device operation for the first ≈0.5 h (**Figure** **5**a), while the same report observed with EL mapping that the luminance progressively became more spatially homogenous for up to 29 h (Figure 5b).^[^ ^99^ ^]^ The study proposed a mechanism for the positive aging effect where electron injection and accumulation in the emissive layer occurred first, and subsequently enhanced hole injection through Coulomb interactions, leading to an increased exciton formation and EL emission over time under constant current injection.^[^ ^99^ ^]^ On the other hand, rapid positive aging can also occur after continuous current density‐voltage sweeps which take seconds to minutes. The cause of this “warming‐up” effect has been attributed to the filling of shell traps by current injection in gradient‐alloyed QDs.^[^ ^102^ ^]^ While this “warming‐up” was studied on green CdZnSeS/ZnSe QD‐LEDs,^[^ ^102^ ^]^ the proposed mechanism may be universal regardless of QD composition, but whether the phenomenon is relevant for blue QD‐LEDs should be verified.

Blue QD‐LEDs may experience more severe positive aging effects during storage compared to red and green devices.^[^ ^34^, ^103^ ^]^ One comparison on CdZnSeS/ZnS blue QD‐LEDs observed a 2.6 fold EQE improvement after 8 days of shelf storage positive aging (Figure 5c), relative to the 1.3‐ and 1.25‐fold increase for red and green devices in the same study, respectively.^[^ ^34^ ^]^ Aside from a few reports,^[^ ^34^, ^103^ ^]^ there is a general lack of systematic studies that compare whether blue QD‐LEDs exhibit more pronounced performance enhancements from positive aging than red and green devices.

While positive aging during storage has been attributed to many different mechanisms, the general understanding currently is that the phenomenon originates from the ETL/cathode half of the device, particularly for QD‐LEDs that use ZnO and ZnMgO ETLs. One common explanation involves volatile acid‐containing encapsulation resins.^[^ ^74^, ^104^, ^105^ ^]^ A comparison of the major components of typical encapsulation resins using gas chromatography–mass spectroscopy measurements detected acrylic acid, *N,N*‐dimethylacrylamide, and isobornyl acrylate.^[^ ^105^ ^]^ Among these, the report further elucidated that acrylic acid may have contributed to the positive aging effect.^[^ ^105^ ^]^ Acrylic acid was proposed to have diffused into the ETL and passivated the surface trap states of ZnMgO to reduce exciton quenching (Figure 5d).^[^ ^74^, ^105^ ^]^ Acrylic acid was also suggested to react with the hydroxyl groups on ZnMgO and CO_2_ in the atmosphere to form zinc carboxylate which passivated the ZnMgO/QD interface.^[^ ^105^ ^]^

Competing explanations for the positive aging during storage propose mechanisms unrelated to the encapsulation resin and/or acrylic acid. For example, an alternative explanation attributed the reaction of the Al cathode with metal oxide ETLs to form oxygen vacancies and AlO_x_.^[^ ^34^, ^106^ ^]^ One report on blue CdZnSeS/ZnS QD‐LEDs monitored the EQE increase with different cathode materials and linked positive aging with the formation of AlO_x_ and oxygen vacancies, which led to a reduced contact resistance and suppressed exciton quenching at the Al/ZnMgO interface (Figure 5e).^[^ ^34^ ^]^ Another explanation for the positive aging effect involves a gradual ripening process of ZnMgO nanocrystals as observed using grazing‐incidence X‐ray scattering techniques, and the improved size uniformity created a more homogenous energy profile with reduced trap states (Figure 5f).^[^ ^100^ ^]^ On the other hand, a systematic study on blue CdZnSeS/ZnS QD‐LEDs decoupled the contributions to positive aging that occurred gradually over 51.5 h to increase the device EQE from 1.72% to 12.58%.^[^ ^73^ ^]^ The report proposes that the gradual improvement in electron injection and a reduction in leakage currents in ZnMgO were the dominant contributors to the positive aging effect, whereas passivation by encapsulation resin products played a minor role.^[^ ^73^ ^]^ Notably, the study's proposed mechanism does not involve the formation of AlO_x_.^[^ ^73^ ^]^ A more recent report on QD‐LEDS of all three primary colors (red, green blue) attributed positive aging to a n‐doping of ZnMgO by reducing hydrogen radicals formed from the reaction of water vapor and Al.^[^ ^103^ ^]^ Notably, acrylic acid resins may also generate reducing hydrogen species.^[^ ^103^ ^]^ The report still noted that future studies are required to completely elucidate the positive aging mechanisms. More broadly, all these findings show that the exact nature of the positive aging effect – both during initial operation or device shelf storage – is complex and not fully understood but may likely be related to multiple synergistic mechanisms. However, separate studies utilize different QD materials, transport layers, and devices structures, which may contribute to the seemingly conflicting results reported across the literature. In addition, positive aging may cause non‐uniform EL with localized intensity fluctuations across the device emission area. To ensure accurate evaluation of performance metrics such as EQE and operational lifetime, the full emission area must be captured, rather than spectrometers or photodetectors that probe only a localized area. This issue becomes more critical for devices with larger pixel areas, where spatial inhomogeneity may be more pronounced.^[^ ^107^ ^]^

### 3.4 Emission Broadening and Low Color Purity

One of the key advantages of QD‐LED technology is its potential to achieve a wide color gamut coverage, making color purity a crucial metric for ensuring high visual quality and color accuracy in advanced display applications. High color purity, characterized by a narrow emission linewidth, for example a FWHM of <20nm, enables vivid and saturated colors with minimal spectral overlap. In contrast, low color purity with a broader FWHM can cause color blending between adjacent subpixels which reduces contrast and may necessitate the use of color filters which adds cost and complexity for practical applications.

Among blue QD emitters, ZnSeTe QDs have received particular attention in recent years as a heavy metal‐free material with the potential for pure‐blue emission. However, in addition to showing limited device stabilities,^[^ ^19^, ^31^, ^46^, ^108^ ^]^ controlling the color purity of the PL of ZnSeTe materials has been an ongoing focus of study. Researchers have looked to alloy ZnSe (bulk bandgap ≈ 2.7 eV) with a small amount of ZnTe (bulk bandgap ≈ 2.25 eV) to tune the center wavelength of the ZnSeTe QD excitonic emission to the pure‐blue range.^[^ ^109^ ^]^ However, the impact of Te‐doping on QD photophysics is complex, and undesirable spectral broadening induced by Te‐doping has received considerable attention. Potential sources of the spectral impurity may include trap emission due to increased lattice disorder, emission from Te‐cluster electronic states, and inhomogeneous broadening due to non‐uniform Te distribution between QDs.

**Figure 6:**
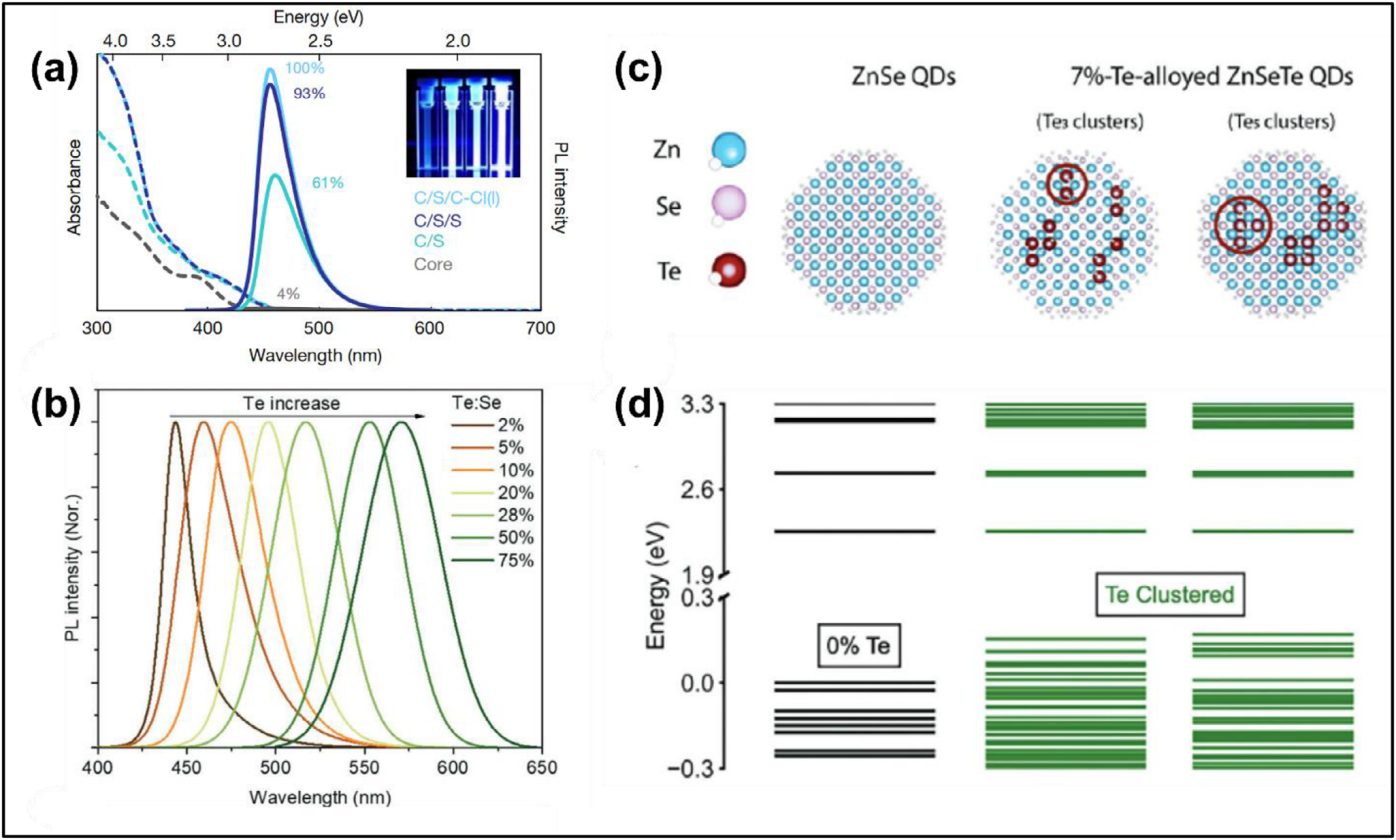
a) Absorption and PL spectra of ZnSeTe‐based QDs with 7% Te content. Spectra are shown for core‐only QDs (grey), core‐shell QDs overcoated with ZnSe (cyan), core‐shell‐shell QDs overcoated with ZnSe followed by ZnS (dark blue), and the core‐shell‐shell QDs with a ZnCl_2_ surface ligand treatment (light blue). Reproduced with permission.^[^ ^15^ ^]^ Copyright 2020, Nature Publishing Group. b) Evolution of the PL spectrum of ZnSeTe/ZnSe/ZnS QDs with increasing Te content. A red shoulder is prominent in lightly doped samples, while the most heavily doped samples exhibit more symmetric, but still broad, PL spectra. Reproduced with permission.^[^ ^110^ ^]^ Copyright 2023, Wiley‐VCH. c) Model QD structures on which DFT calculations were performed, yielding d) the corresponding band‐edge energy levels. Te clustering introduces additional states below the valence band maximum. Reproduced with permission.^[^ ^46^ ^]^ Copyright 2023, Wiley‐VCH.

ZnSeTe QDs have been observed to exhibit broadened PL linewidths, particularly at higher Te contents. Among ZnSeTe QDs with emission peaks in the “blue” range (450–495 nm), the narrowest reported ensemble PL FWHMs are approximately ≈22 nm.^[^ ^19^, ^46^ ^]^ However, in other prominent reports they have been much broader, where their best achieved FWHM is 35 nm.^[^ ^15^, ^33^ ^]^ An additional characteristic of many ZnSeTe QDs’ emission spectra is a pronounced red tail which can extend to green wavelengths (**Figure** **6**a), although its shape and extent vary significantly between syntheses and the content of Te relative to Se (% Te). In general, at low Te contents, it may be difficult to distinguish between a genuine redshift of the QD bandgap due to conventional alloying in contrast with increased emission from a red, non‐excitonic tail. At higher Te contents, the ZnSeTe PL generally grows more symmetric, losing the red shoulder, and may further broaden (Figure 6b). The prominence and spectral range of this red shoulder in lightly doped QDs were also observed to vary significantly depending on the reactivities of the Se and Te precursors used in the core synthesis. These tests were performed for a fixed absolute Te content,^[^ ^19^ ^]^ indicating that the tail emission mechanism is highly dependent on the core morphology.

In many colloidal QDs, a red shoulder is often associated with the radiative recombination of localized trap sites. ZnTe has a larger lattice constant (≈6.1 Å) relative to ZnSe (≈5.7 Å), and due to this mismatch the introduction of Te may contribute to interfacial defects and stacking faults.^[^ ^15^, ^19^, ^31^ ^]^ Many researchers have developed synthetic strategies for reducing the number of trap sites and improve the QD optical properties. A study in 2019 pioneered a core/shell/shell ZnSeTe/ZnSe/ZnS architecture to target interfacial trap sites.^[^ ^31^ ^]^ Introduction of a ≈1 nm ZnSe inner shell redshifted the center emission wavelength of a 2% Te sample from 437 to 441 nm. It also lowered the PL FWHM from 40 to 32 nm, consistent with the reduction in shallow interfacial traps. Despite the apparent reduction in trap density, the PL spectrum was still asymmetric, however, with a prominent red tail suggestive of some form of trap emission.

Multiple studies have looked to reduce the number of lattice defects in ZnSeTe QDs (and generally improve the particles’ optical properties) with the addition of HF during nanocrystal growth.^[^ ^15^, ^33^, ^46^ ^]^ In one work, researchers added HF during the growth of the ZnSe mid‐shell, which they argued removed bulky organic ligands that hindered epitaxial shell growth surface and etched away oxidized defect sites. The resulting 7% Te QDs showed a near‐unity PLQY, consistent with the elimination of deep, nonradiative traps.^[^ ^15^ ^]^ However, the PL FWHM was 36 nm and the sample exhibited a prominent red shoulder in its emission.

A subsequent report from the same group closely studied the influence of HF on the optical properties of ZnSeTe QDs. In this work, the researchers synthesized a batch of ZnSeTe/ZnSe/ZnS QDs containing 3% Te with peak emission at 447 nm. While the samples showed very narrow PL FWHMs of ≈14 nm.^[^ ^33^ ^]^ it is worth noting that the emission peak fell outside of the “blue” range due to the very slight Te content. The authors added HF during the initial ZnSe shell growth of these samples, allowing the HF to act on the ZnSeTe core and ZnSe shell interfaces. Transmission electron microscopy images showed that the addition of HF favored the growth of well faceted cube‐shaped core‐shell structures. Optically, the addition of HF generally improved the samples’ PLQYs (with the highest measured at 97%). For the highest amount of HF added, the PL spectrum was considerably narrowed at 100K. However, the samples’ room temperature spectra were largely the same across the HF series, with a slight but consistent red tail. These results imply that, for this sample, the addition of HF eliminated surface trap sites that diminished the PLQY, but these traps did not significantly contribute to the emission at room temperature and were not responsible for the lingering spectral broadening.

The persistence of a red shoulder despite extensive synthesis efforts to optimize PLQY and reduce the number of trap sites suggests that this red shoulder should not be exclusively attributed to conventional trap emission. Indeed, many researchers have inferred that emission from mid‐gap states associated with clusters of Te (Figure 6c) also plays a key role. This behavior has been observed in bulk ZnSe_1‐x_Te_x_ alloys, with Te atoms or clusters of multiple Te atoms giving rise to hole‐localizing states on the order of hundreds of meV below the valence band maximum.^[^ ^111^, ^112^, ^113^, ^114^ ^]^ In ZnSeTe QDs, multiple studies have found that the red tail in ZnSeTe has a slower radiative lifetime than the primary component in the spectrum. The spectral position of the red shoulder is also less sensitive to temperature than the primary spectral component.^[^ ^33^, ^108^ ^]^ Both of these observations are consistent with the red emission stemming from a distinct, non‐excitonic mechanism, and density functional theory calculations (Figure 6d) have supported assigning these transitions to Te‐cluster states.^[^ ^46^, ^108^ ^]^

Previous spectroscopic studies of CdSe nanomaterials doped with small amounts of Te provide additional evidence of these mid‐gap Te cluster states.^[^ ^115^, ^116^, ^117^ ^]^ In one report, researchers observed that CdSe QDs doped with 3–7% Te exhibited large and positive (that is, less stable) biexciton binding energies on the order of hundreds of meV. This result is consistent with the two holes of the biexciton localizing on a Te defect state, increasing the Coulombic repulsion between the delocalized electrons.^[^ ^117^ ^]^ Greater non‐radiative Auger recombination of the biexciton was also observed in Te‐doped CdSe nanoplatelets, which was attributed to increased hole‐hole overlap on cluster sites.^[^ ^116^ ^]^ These studies lend credence to the existence of hole‐localizing cluster states apart from conventional trap sites in Te‐doped QDs, although additional spectroscopic studies are required to reveal the properties of Te clusters in the particular case of ZnSeTe QDs.

The non‐uniform distribution of Te between different QDs also likely broadens the spectral linewidth in ZnSeTe QDs, but it appears to play a greater role in more highly doped samples. Consistent with other reports,^[^ ^15^, ^31^ ^]^ one careful study found that highly doped (>20% Te) QDs generally exhibited ensemble PL spectra that were broadened but more symmetric relative to a lightly doped 2% Te sample.^[^ ^108^ ^]^ The authors characterized a 20% Te sample, which had an emission peak at ∼495 nm with a FWHM of ∼38 nm. Wavelength‐ and time‐resolved photoluminescence measurements on this sample showed that the lifetime was largely constant across emission wavelengths. This result suggests that there is a similar emission mechanism across the entire spectrum. Moreover, for the same sample, photoluminescence excitation (PLE) measurements implied that different regions of the PL spectrum corresponded to sample populations with distinct absorption profiles. Transmission electron microscopy measurements suggested that the sample had high size monodispersity, so this heterogeneity likely stems from variation in the number and distribution of Te atoms per QD. As a result, the bandgaps of different QDs may be redshifted to different degrees due to a conventional alloying effect – or, possibly, different QDs may have distinct Te cluster motifs, forming mid‐gap states with varying energies relative to the exciton.

Overall, the optical properties of ZnSeTe QDs certainly merit further study. One open question is what fraction of ZnSeTe QDs emit from a Te cluster state relative to an excitonic state or a conventional trap state, or some combination thereof. Another open question concerns the number and spectral range of possible Te cluster states that can exist within a given QD, or across a QD ensemble. An in‐depth study of ZnSeTe QDs using single‐particle spectroscopy, particularly at liquid‐helium temperatures, could provide valuable information regarding the properties of Te‐cluster emission. Such studies could, in turn, inform synthetic efforts to achieve narrow emission from ZnSeTe QDs in the pure blue emission range.

## 4 Recent Advances in State‐of‐the‐art Blue QD‐LEDs

### 4.1 Synthesis and Design of Tailored Core/Shell Heterostructures

To achieve efficient pure blue QD‐LEDs, the QDs composing the emitting layer must be synthetically optimized through selection of non‐toxic candidate materials, improvement of synthetic routes, and rational design of advanced core/shell heterostructures. The ideal blue‐emitting QD material should demonstrate excellent optical properties including near‐unity PLQY and a narrow emission spectrum at 467 nm, in addition to high stability under optical and electric excitation and long‐term shelf storage. Recent synthetic efforts aimed at optimizing blue QDs to meet these criteria are essential for the development of commercially viable blue QD‐LEDs and are discussed here.

**Figure 7:**
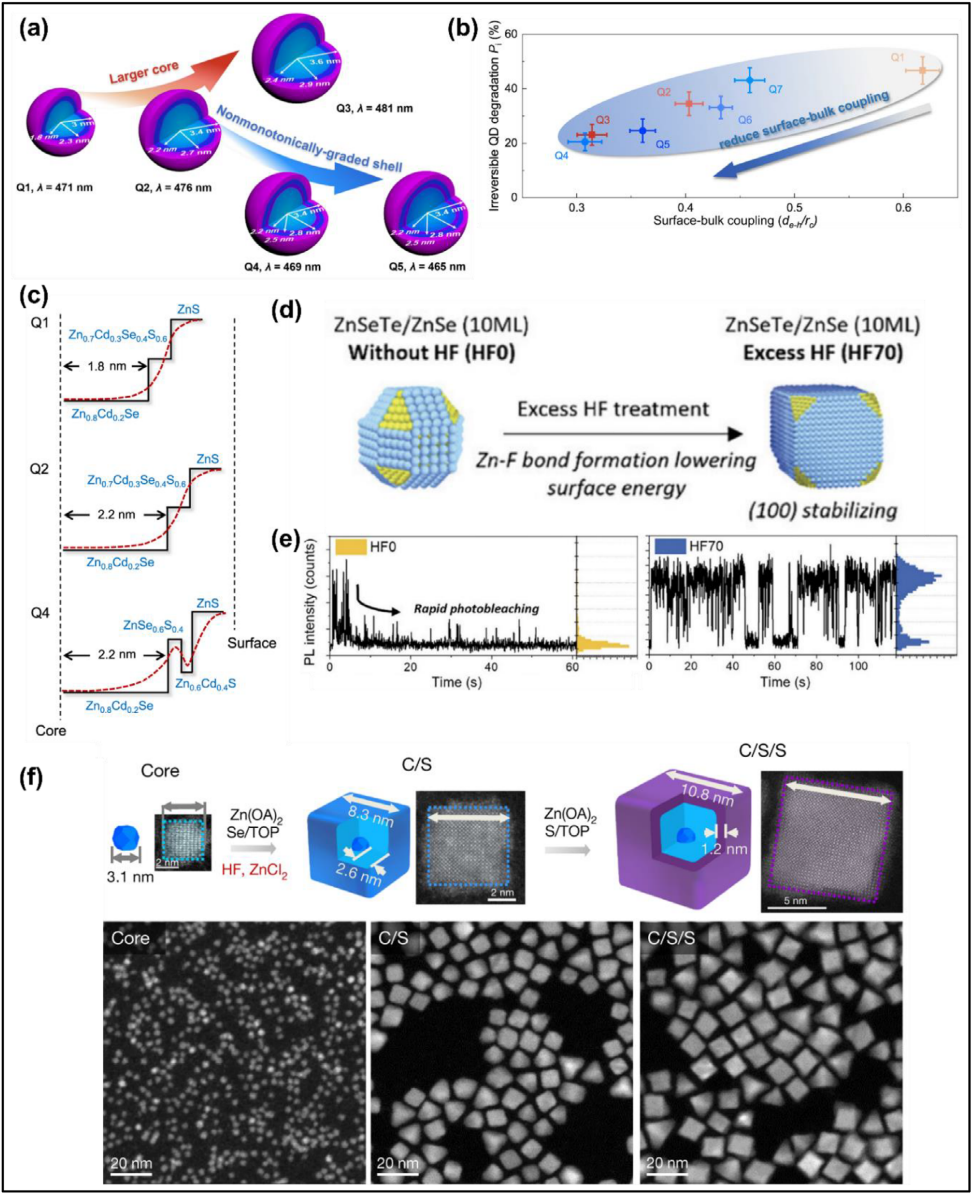
a) Schematic illustrating strategies to enhance Cd‐based blue QDs by increasing the core size or engineering a non‐monotonic shell structure, b) correlation between the QD degradation rate and the degree of surface‐bulk coupling, and c) CBM change of the QDs as the core size is increased or by introducing a non‐monotonic intermediate shells. Reproduced with permission.^[^ ^22^ ^]^ Copyright 2023, Nature Publishing Group. d) Schematic illustrating the growth and shape change of ZnSeTe/ZnSe QDs with or without the use of HF during synthesis, and e) single‐dot PL intensity with time and intensity histogram of the ZnSeTe/ZnSe QDs with or without the use of HF during synthesis. Reproduced with permission.^[^ ^33^ ^]^ Copyright 2024, Wiley‐VCH. f) Schematic illustrating the synthesis of the ZnSeTe core (left), ZnSeTe/ZnSe core/shell (middle), and ZnSeTe/ZnSe/ZnS core/shell/shell (right) QDs, and their corresponding TEM images (bottom row). Reproduced with permission.^[^ ^15^ ^]^ Copyright 2020, Nature Publishing Group.

CdSe‐ and CdS‐based blue QDs with small core sizes have high surface‐to‐volume ratios and are more susceptible to the influence of traps and surface states. Increasing the core size and/or creating a non‐monotonic energy level are viable strategies to improve exciton confinement to the QD core (**Figure** **7**a,b). Giant CdZnSeS QDs can be synthesized with a Se‐rich center and S‐rich outer core and a graded Zn content increasing from the center to the outer surface.^[^ ^57^ ^]^ The composition gradient reduced core/shell lattice strain and created a non‐monotonic CBM change, which improved exciton confinement and suppressed Auger recombination.^[^ ^57^ ^]^ Blue‐emitting QD‐LEDs based on the giant CdZnSeS/ZnS QDs show high EQEs of 24%, EL emission at 479 nm, maximum luminance of 57000 cd m^−2^, and a T_50_ lifetime of 27000 h at 100 cd m^−2^.^[^ ^57^ ^]^ Another report increased the core size to reduce surface‐bulk coupling of ZnCdSe/ZnCdSeS/ZnS QDs (core diameter: 2.4 nm), and demonstrated one of the most stable blue‐emitting QD‐LEDs reported, with a T_50_ lifetime of 80377 h at 100 cd m^−2^, EL emission at 482 nm, and EQE of 20.4%.^[^ ^22^ ^]^ The same study also demonstrated a different QD heterostructure design with non‐monotonically‐graded ZnCdSe/ZnSeS/ZnCdS/ZnS QDs with pure blue EL emission at 467 nm, narrow linewidth of 20 nm, and EQE of 20.1%. The wider bandgap ZnSeS inner sub‐shell introduced a non‐monotonic energy barrier (Figure 7c) that better confines excitons to the QD core, and the ZnCdS outer sub‐shell reduced the charge injection barrier width without inducing lattice strain.^[^ ^22^ ^]^ Separately, blue‐emitting ZnCdSe/ZnS//ZnS QDs with a 472 nm PL emission (31 nm FWHM) were synthesized with double ZnS shells via a low temperature nucleation and high temperature shell growth method.^[^ ^56^ ^]^ The double shell provided effective surface passivation and suppressed blinking, with >98% “ON” time and increased PLQY to 92%.^[^ ^56^ ^]^

Binary ZnSe QDs have been considered as candidate blue emitters. Bulk ZnSe has a suitable bandgap (≈2.7 eV) for pure blue emission, but quantum‐confined ZnSe QDs emit in the violet region (<430 nm). Ongoing research efforts aim to improve synthesis for high‐quality ZnSe QDs with larger cores to shift the emission wavelength into the blue. One study synthesized bulk‐like ZnSe/ZnS QDs with a large 10 nm core diameter, thin (≈1 nm) ZnS shell, and attained 95% PLQY.^[^ ^47^ ^]^ The devices exhibited EL emission at 445 nm with a narrow linewidth (12 nm FWHM) and EQE of 12.2%.^[^ ^47^ ^]^ To further redshift the EL emission into the pure blue range (460 – 475 nm), even larger ZnSe nanocrystals (core diameter: 35 nm) were synthesized through sequential injection of high‐reactivity and low‐reactivity Zn and Se precursors.^[^ ^45^ ^]^ The ZnSe/ZnS nanocrystals show PL emission between 455 and 470 nm and decent PLQY (60%), but no device results were reported.^[^ ^45^ ^]^

In addition to varying the core size, alloying the ZnSe core with Te atoms – producing ternary ZnSeTe QDs – also tunes the bandgap and influences other key properties. As we discussed in the context of emission purity, in Section 3.4, the addition of hydrofluoric acid (HF) dramatically influences the optical behavior of the ternary QDs. Te is highly sensitive to oxygen, and the oxidized species constitute trap states that cause non‐radiative recombination.^[^ ^46^ ^]^ The HF is effective in removing these oxidized species and other defects and from the ZnSeTe during shell growth.^[^ ^15^, ^33^ ^]^ As a result, the addition of HF can significantly improve the PLQY of the QD sample.^[^ ^31^, ^33^, ^46^ ^]^ In addition to bolstering the PLQY, the addition of an appropriate amount of HF can inhibit photobleaching, leading to improved intensity stability. Again, this effect is believed to stem from reduced lattice disorder at the core/shell interface (Figure 7d).^[^ ^33^ ^]^ In this study, single‐QD PL measurements were used to observe that ZnSeTe/ZnSe/ZnS QDs synthesized without HF exhibited rapid and irreversible photobleaching within a few seconds upon PL excitation. In contrast, using an optimized HF amount in the synthesis suppressed defect formation and led to reversible blinking characteristics, a higher ON‐time ratio, and reduced spectral diffusion for the ZnSeTe/ZnSe/ZnS QDs (Figure 7e).^[^ ^33^ ^]^ In another study, TEM images showed that combining both HF and ZnCl_2_ reduces the number of stacking faults and promotes the growth of the (100) facet. The resulting QDs were cubic shaped ZnSeTe/ZnSe/ZnS QDs with a high PLQY of 93% (Figure 7f).^[^ ^15^ ^]^ Given the highly toxic nature of HF, HF‐free ZnSeTe synthesis has also been developed and achieved decent device performance.^[^ ^46^, ^118^, ^119^ ^]^ One strategy introduced excess ZnCl_2_ without HF before ZnSe shell growth, and reported ZnSeTe/ZnSe/ZnS QDs with 94% PLQY.^[^ ^118^ ^]^ Another method uses alternative fluoride‐containing precursors such as ammonium fluoride (NH_4_F)^[^ ^119^ ^]^ or benzenecarbonyl fluoride^[^ ^46^ ^]^ to replace HF. NH_4_F‐assisted synthesis achieved ZnSeTe/ZnSe/ZnS QDs with 95% PLQY, PL emission wavelength between 450 and 465 nm, and the QD‐LED device reached 17.2% EQE.^[^ ^119^ ^]^

For ZnSeTe/ZnSe/ZnS QDs, the wide bandgap ZnS outer shell passivates surface defects and confines excitons within the QD. Controlling the ZnSe and ZnS thickness is important to maximize the QD‐LED EL properties. Increasing shell thickness generally leads to a higher PLQY, redshifted emission, and narrower linewidth due to more effective trap passivation, but excessive shell thickness hinders charge injection and reduces PLQY.^[^ ^32^, ^120^ ^]^ A study reported varying the ZnSe shell thickness from 1.9 to 3 nm, and observed a redshifted EL emission from 452 to 456 nm and decreased linewidth from 33 to 22 nm.^[^ ^32^ ^]^ The device EQEs were 11% (1.9 nm ZnSe shell thickness), 18% (2.5 nm), and 13.4% (3 nm), which illustrates the importance of controlling the ZnSe shell thickness to tune the QD‐LED EL characteristics. While these studies have explored the impacts of shell structure and thickness on device performance, the direct relationships between the QD shells and QD‐LED operational stability require further study, especially given that the shell properties will play a critical role in influencing charge balance in blue QDs.

Te aggregation may lead to stacking faults, asymmetric spectral broadening, and poor lattice stability for ZnSeTe QDs as discussed in previous sections. A recent breakthrough to suppress Te aggregation was reported^[^ ^19^ ^]^ for quaternary ZnSeTeS QD synthesis using sulfur coordinated triphenyl phosphite (TPP‐S) precursor. Highly reactive TPP‐S balances the reactivity between the anion precursors, and the electronegativity of S promotes carrier delocalization of Te and prevents their aggregation, resulting in homogeneous ZnSeTeS QDs (3% sulfur content) with reduced stacking faults and enhanced structural stabilization compared to the reference ZnSeTe QDs.^[^ ^19^ ^]^ The ZnSeTeS/ZnSe/ZnS devices showed pure blue EL emission at 460 nm and EQE of 24.7%, and the lower energy tail emission was eliminated resulting in a narrow linewidth of 17 nm. The T_50_ operational lifetime was 30000 h at 100 cd m^−2^, which is among the highest reported for Cd‐free blue QD‐LEDs.^[^ ^19^ ^]^ This represents a promising path forward toward heavy metal‐free QD‐LEDs with operational stability that rival the best‐reported for Cd‐containing^[^ ^22^ ^]^ QD‐LEDs (T_50_ of ≈80000 h at 100 cd m^−2^ for ZnCdSe/ZnCdSeS/ZnS).

### 4.2 Ligand Exchange and Effective Surface Passivation

**Figure 8:**
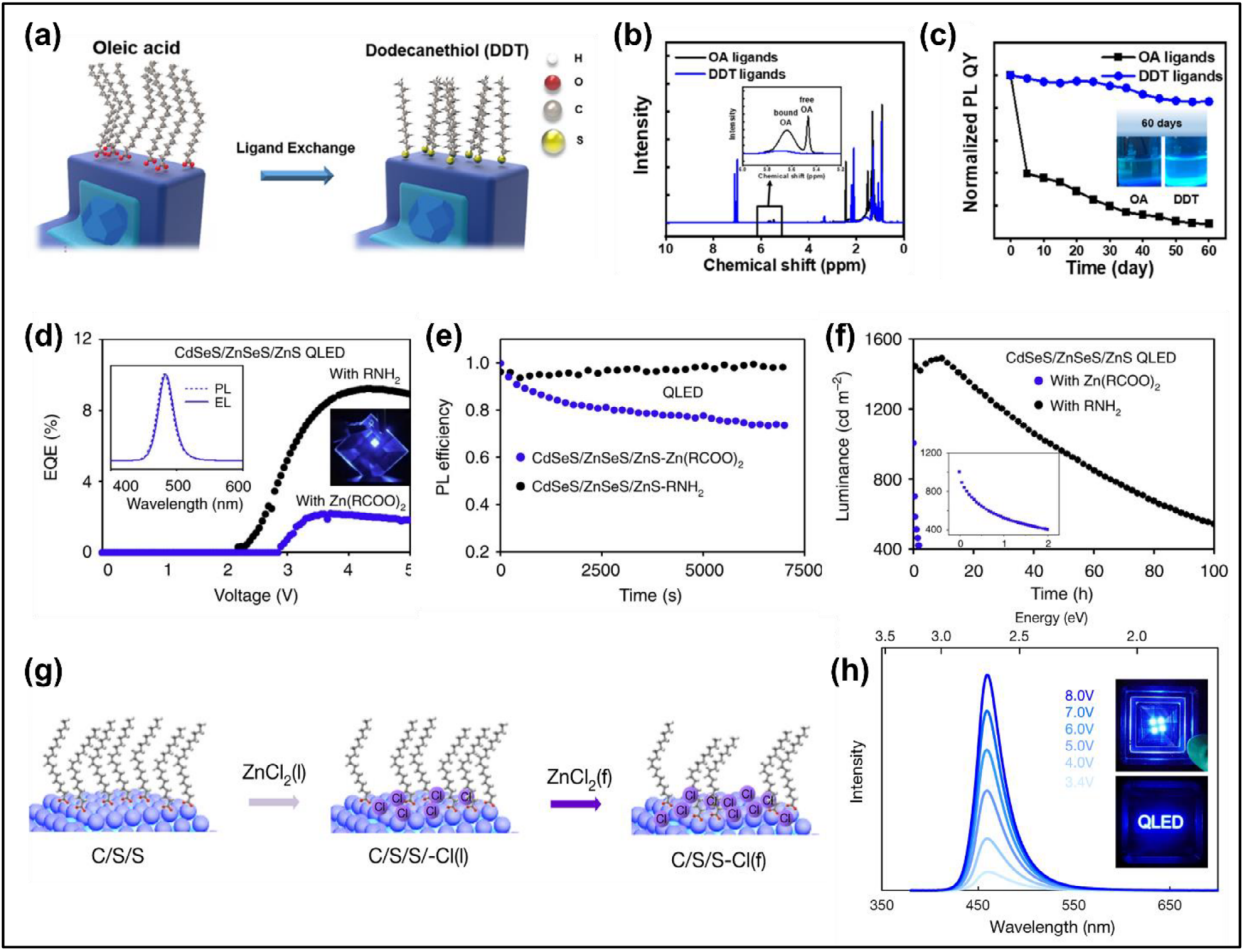
a) Schematic showing the ligand exchange of oleic acid to 1‐dodecanethiol for ZnSeTe/ZnSe/ZnS QDs, b) ^1^H NMR spectra before and after ligand exchange, and c) comparison of the ambient air PLQY stability of the QDs with oleic acid or 1‐dodecanethiol ligands. Reproduced with permission.^[^ ^118^ ^]^ Copyright 2022, American Chemical Society. d) EQE of the blue CdSeS/ZnSeS/ZnS QD‐LEDs with either carboxylic acid ligands or amine ligands, e) PL efficiency change of the QD‐LEDs driven at a constant current density of 100 mA cm^−2^, and f) operational lifetime testing of the QD‐LEDs in constant current mode. Reproduced with permission.^[^ ^64^ ^]^ Copyright 2020, Nature Publishing Group. g) Schematic of the oleic acid to ZnCl_2_ two‐step ligand exchange process, first during QD colloidal synthesis, and second as a washing treatment on the QD thin‐film, and h) voltage‐dependent EL spectra of the target QD‐LED. Reproduced with permission.^[^ ^15^ ^]^ Copyright 2020, Nature Publishing Group.

Ligand exchange is often applied to substitute the native surface ligands of as‐synthesized colloidal QDs. For device applications, shorter and less insulating ligands are desirable to reduce interparticle distance and improve charge injection and transport. In this regard, thiol ligands are popular because of their shorter chain length, stronger binding to the QD surface, and higher resilience to surface desorption during device operation. Common choices are the alkanethiols including 1‐dodecanethiol and 1‐octanethiol.^[^ ^118^, ^121^, ^122^ ^]^ The use of shorter 1‐octanethiol to substitute native oleic acid ligands improved charge balance for blue‐violet ZnCdS/ZnS QDs and achieved 12.2% EQE, with an EL emission peak of 443 nm (21.5 nm FWHM).^[^ ^121^ ^]^ This was the first reported blue‐violet QD‐LED that broke the 10% efficiency barrier in 2015.^[^ ^121^ ^]^ Ligand exchange is also important for QD‐LED operational lifetime by replacing thermally and electrochemically unstable ligands with more robust alternatives. Ligand exchange of oleic acid with 1‐dodecanethiol (**Figure** **8**a,b) improved the oxidation stability of blue ZnSeTe/ZnSe/ZnS colloidal QDs, which maintained >80% PLQY after 60 days of storage under ambient conditions (Figure 8c).^[^ ^118^ ^]^ The improved ambient air stability was attributed to the stronger binding of 1‐dodecanethiol with the QD which prevented ligand oxidation and desorption.^[^ ^118^ ^]^ Although thiol ligands have stronger binding to the QD surface as compared to carboxylic acids, there are concerns that thiol ligands are still electrochemically reactive.^[^ ^65^ ^]^ In contrast, organic amines and neutral organophosphines may be fully inert electrochemically for device applications.^[^ ^64^, ^97^ ^]^ One report observed that although ligand exchange of carboxylic acid ligands with oleylamine reduced the PLQY of blue‐emitting CdSeS/ZnSeS/ZnS QDs from 80% to 60%, the QD‐LED EQE was enhanced significantly to ≈10% (Figure 8d) compared to ≈2% EQE for the control device.^[^ ^64^ ^]^ The target device T_50_ lifetime was 10000 h at 100 cd m^−2^ which was attributed to the more electrochemically robust organic amine ligands (Figure 8e,f).^[^ ^64^ ^]^

Metal halide MX_2_ ligands may be favored over bulky organic ligands because the smaller metal halides are less sterically hindered, achieving better surface coverage and trap passivation. Halide ligands also exhibit stronger binding with Zn dangling bonds compared to carboxylic acids. This is exemplified by ZnX_2_ ligand exchange for ZnSeTe QDs.^[^ ^15^, ^118^, ^123^ ^]^ Specifically,^[^ ^15^ ^]^ a two‐step ZnCl_2_ ligand exchange process was applied to replace native oleic acid; first in liquid‐phase after QD colloidal synthesis, and second as a washing treatment on the QD thin‐film (Figure 8g). Effective ligand exchange and trap passivation with ZnCl_2_ led to unity PLQY for ZnSeTe/ZnSe/ZnS QDs with a 457 nm peak emission wavelength (36 nm FWHM), and also increased hole mobility by an order of magnitude to achieve better charge injection balance. The ZnSeTe/ZnSe/ZnS devices (Figure 8h) demonstrated an EQE of 20.2%, which was the first reported blue QD‐LED that exceeded 20% EQE, and T_50_ lifetime of 15850 h at 100 cd m^−2^.^[^ ^15^ ^]^ Despite the impressive results, the electrochemical stability of ZnCl_2_ passivation is less understood, with some reports suggesting that the surface binding energy may be relatively weak.^[^ ^124^ ^]^ It is also necessary to develop a scalable version of the washing process for ligand exchange, to replicate its effectiveness when applied to large‐area patterned devices, such as those fabricated via inkjet printing.

Surface ligands can be used to tune the band alignment and energy levels of the QD in the device stack. This presents a versatile strategy to control charge injection and charge balance into the QD. The vacuum energy level at the QD surface can be controlled by the combined contributions of the ligand intrinsic dipole moment and ligand‐surface bond formation.^[^ ^125^, ^126^ ^]^ While this has been applied successfully for high performance QD solar cells,^[^ ^127^, ^128^ ^]^ the strategy has been less discussed for QD‐LEDs. As an example, solid‐state ligand exchange of tetrabutylammonium iodide with 1,2‐ethanedithiol (EDT) for PbS QD solar cells downshifted the vacuum level by 0.39 eV and upshifted the CBM by 0.19 eV.^[^ ^127^ ^]^ This effectively facilitated hole transport to the anode while blocking electron transfer, leading to a certified world‐record 8.55% efficiency QD solar cell.^[^ ^127^ ^]^ Finally, the performance and stability of pixelated QD‐LEDs is critically affected by the morphology, uniformity, and thickness of the patterned QD film. The chemical structure, chain length, and dipole moment of surface ligands will determine the QD dispersity and wettability in ink solvents, which is critical for the QD film formation process during patterning.^[^ ^129^, ^130^ ^]^

### 4.3 Transport Layers with Efficient Charge Injection and Ideal Energy Level Alignments

Confining the exciton recombination zone within the emissive layer is crucial for high performance QD‐LEDs. The ideal HTL and ETL combination needs to simultaneously balance charge transport and maximize carrier injection into the emissive layer. Transport layer improvement strategies can be generally categorized into 1) increasing charge carrier mobility, 2) controlling band alignment for efficient majority carrier injection while blocking minority carrier leakage, and 3) reducing trap states and structural disorder.

Various p‐type materials have been explored as HTLs, with common selections including CBP (4,4′‐bis(9‐carbazolyl)‐1,1′‐biphenyl), PVK (poly(9‐vinlycarbazole)), poly‐TPD (poly(4‐butyl‐*N*,*N*‐diphenylaniline)), TCTA (4,4′,4′′‐tri‐9‐carbazolyltriphenylamine), and inorganic metal oxides such as nickel oxide (NiO_x_) and molybdenum oxide (MoO_x_). Broadly speaking, each of these materials may have specific drawbacks that limit its widespread utilization, such as low hole mobility, electrochemical and thermal instability, or poor solvent compatibility and wetting with QD deposition. Metal oxides typically have good thermal and material stability, but their hole conductivity is relatively low, which has given rise to the prevalence of organic and polymer HTLs in current QD‐LEDs. Recent state‐of‐the‐art blue QD‐LEDs have mostly converged on two choices: TFB and PF8Cz (Poly((9,9‐dioctylfluorenyl‐2,7‐diyl)‐alt‐(9‐(2‐ethylhexyl)‐carbazole‐3,6‐diyl))). Both are conjugated co‐polymers that have the dioctylfluorene unit in common, but TFB has a triphenylamine unit, while PF8Cz features a fused ring carbazole unit. Both materials have similar hole mobilities (≈10^−3^ cm^2^ V^−1^ s^−1^) and HOMO energies (−5.4 eV), but the advantage of PF8Cz is its reduced energetic disorder, with a more narrow HOMO distribution and shallower LUMO level (−2.3 eV) compared to TFB (−2.6 eV). Consequently, PF8Cz can more effectively block leakage electrons^[^ ^18^ ^]^ and better confine the exciton to the emissive layer. PF8Cz was observed to improve the performance of both green and blue QD‐LEDs compared to the reference based on TFB, with the blue CdZnSe/ZnS devices with PF8Cz achieving 21.9% EQE with a 479 nm EL emission (23 nm FWHM).^[^ ^18^ ^]^ Utilizing PF8Cz as the HTL, blue CdZnSe/ZnS QD‐LEDs demonstrated a T_50_ lifetime^[^ ^18^ ^]^ of 24000 h at 100 cd m^−2^. Despite its limitations, TFB remains a popular HTL choice due to its good reproducibility and wide applicability for different QD materials and device structures. While efficient and stable blue QD‐LEDs have been demonstrated with PF8Cz,^[^ ^18^, ^19^ ^]^ state‐of‐the‐art device performance results have also been achieved using TFB.^[^ ^15^, ^22^ ^]^ More broadly, the best‐reported operational lifetimes across the literature of blue QD‐LEDs with either TFB or PF8Cz are comparable, on the order of ≈10^4^ h at 100 cd m^−2^. These results may be reconcilable if shorter timescale degradation processes currently dominate the instability of blue QD‐LEDs rather than HTL degradation as discussed in previous sections, but this requires further investigation to prove conclusively.

**Figure 9:**
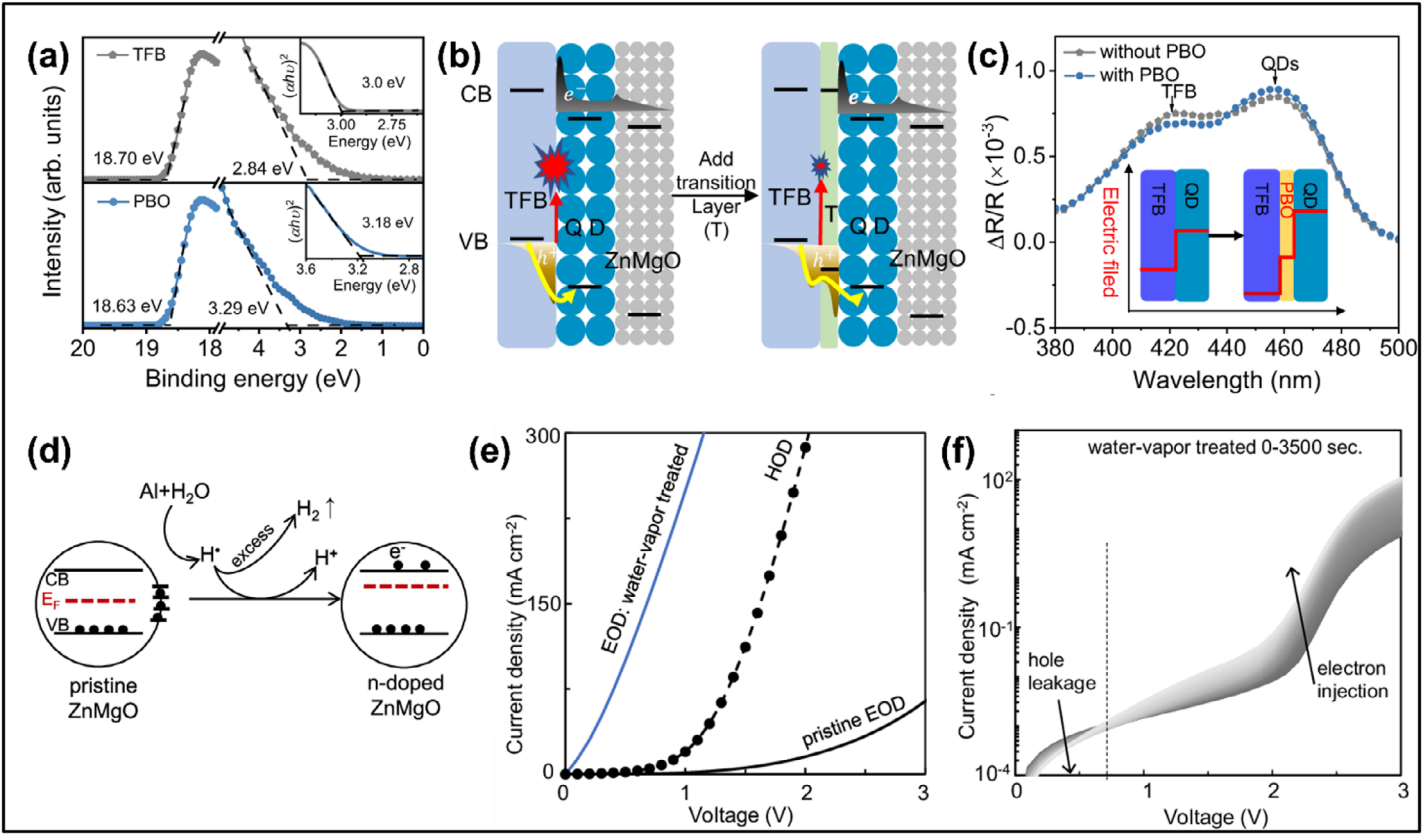
a) UPS spectra of the valence band region and secondary electron cut‐offs of the TFB and PBO films, b) schematic illustrating the PBO interlayer (in green) and the cascading band alignment, and c) electroabsorption spectra for the QD‐LEDs under 0.5 V bias with or without PBO and schematic of the electric field distribution in the device. Reproduced with permission.^[^ ^29^ ^]^ Copyright 2024, Nature Publishing Group. d) Schematic of the in situ reductive doping strategy to n‐dope ZnMgO using the reaction of H_2_O and Al, e) current density change of the single‐carrier devices after the reductive n‐doping of ZnMgO, and f) change in the current density‐voltage characteristics of the devices showing an improved electron injection and suppressed hole leakage. Reproduced with permission.^[^ ^103^ ^]^ Copyright 2025, Nature Publishing Group.

Although HTL degradation may not necessarily limit the stability of blue QD‐LEDs currently, HTL development is still important because improved hole injection will likely address other degradation mechanisms, given that excess electron accumulation is thought to be a major challenge for blue devices. Besides designing novel HTL materials, other strategies to improve hole injection into the emissive layer include using bilayer HTLs,^[^ ^131^, ^132^ ^]^ doped HTLs,^[^ ^52^, ^133^ ^]^ or interlayers at the QD/HTL interface to facilitate hole transport.^[^ ^29^ ^]^ For example, poly(p‐phenylene benzobisoxazole) (PBO) has high hole mobility (≈10^−3^ cm^2^ V^−1^ s^−1^) and a deep HOMO level (−5.88 eV, 0.52 eV deeper than TFB) (**Figure** **9**a).^[^ ^29^ ^]^ A 10 nm PBO interlayer inserted in between TFB HTL and CdZnS/ZnS blue QDs (green layer in Figure 9b) formed a cascading band alignment to bridge hole transport into the emissive layer and improve charge balance. The CdZnS/ZnS QD‐LED achieved a peak EQE of 23%, EL emission at 458 nm, and a T_50_ lifetime of 41000 h at 100 cd m^−2^, among the longest lifetimes reported for blue QD‐LEDs.^[^ ^29^ ^]^ Electroabsorption characterizations demonstrated that the stepwise electric field of PBO (Figure 9c) reduced hole accumulation in TFB, improved hole injection, and promoted exciton generation in the emissive layer, which contributed to the improved QD‐LED efficiency and stability.^[^ ^29^ ^]^

ETL choice is more limited for the recent generation of blue QD‐LEDs, with the vast majority of devices using ZnO‐based nanoparticles (including ZnMgO). Alternative n‐type organic materials or metal oxides, in particular TiO_2_ and SnO_2_, have been investigated as ETLs. Although current device results using TiO_2_ and SnO_2_ are not yet competitive with ZnO‐based devices, recent studies have made progress on improving their efficiency and operational lifetime.^[^ ^134^, ^135^, ^136^ ^]^ Given the higher electrochemical stability of TiO_2_ and SnO_2_ and the potential to mitigate the positive aging effect, the materials warrant further development for QD‐LED applications.

ZnO is generally considered an excellent ETL material for its chemical and thermal stability, high electron mobility (>10^−3^ cm^2^ V^−1^ s^−1^), shallow CBM (−4.4 eV), deep VBM (>7.0 eV), and solution processability by orthogonal solvents. The optoelectronic properties of ZnO can be readily tuned by controlling the nanoparticle size,^[^ ^137^, ^138^ ^]^ or metal doping/alloying with Al, Li, Ga, Sn, or Mg.^[^ ^139^, ^140^, ^141^, ^142^ ^]^ Particularly, ternary ZnMgO (typically 10–15% Mg content) has become the ubiquitous choice for state‐of‐the‐art blue QD‐LEDs. Substitutional alloying occurs due to the same charge state (+2) and similar ionic radius between Mg (72 pm) and Zn (74 pm). ZnMgO has a wider bandgap, shallower CBM, and reduced electron conductivity and mobility compared to pure ZnO. Mg doping suppresses the formation of oxygen vacancy and zinc interstitial defects because of the stronger bonding energy of Mg─O (393.7 kJ mol^−1^) compared to Zn‐O (284.1 kJ mol^−1^).^[^ ^70^ ^]^ The reduced trap density mitigates exciton quenching and leads to an increased device EQE.^[^ ^69^, ^70^ ^]^

More broadly, reducing electron injection for blue devices may be a strategy to improve efficiency and stability. Electron injection can be reduced by ETL doping and defect passivation strategies.^[^ ^51^, ^133^ ^]^ Halide passivation of ZnO or ZnMgO with Cl^−^ or F^−^ anions has led to device performance improvements in red and green QD‐LEDs,^[^ ^119^, ^143^, ^144^ ^]^ but the strategy has been less reported for blue devices. Electron injection can also be reduced by inserting an electron‐blocking interlayer at the QD/ETL interface such as poly(methyl methacrylate) (PMMA), *tert*‐butyldimethylsilyl chloride‐modified poly(*p*‐phenylene benzobisoxazole) (TBS‐PBO), or inorganic Al_2_O_3_.^[^ ^13^, ^133^, ^145^ ^]^ For example, insulating PMMA was used as an interlayer between the ZnO ETL and blue ZnCdSe/ZnS//ZnS QDs to impede excess electron injection from the cathode, and single‐carrier device studies indicated that the charge balance improved by 50% with the PMMA layer.^[^ ^56^ ^]^ The blue QD‐LEDs demonstrated an EQE of 16.2% at an EL emission of 479 nm (34 nm FWHM), relative to the 9.8% EQE for the reference device without PMMA interlayer.^[^ ^56^ ^]^ The disadvantage of reducing electron injection is the higher driving voltage and decreasing overall current of the device. Therefore, improving HTL hole injection remains the more desirable option to balance charge injection, although reducing ETL electron injection may be a complementary approach.

A recent report^[^ ^103^ ^]^ proposed that pristine ZnMgO exhibits low conductivity due to insufficient n‐type doping, effectively behaving as an intrinsic semiconductor. An in‐situ reductive doping strategy (Figure 9d) was demonstrated, where the reaction of H_2_O and Al generated reducing hydrogen radicals at the Al/ZnMgO interface and enabled state‐filling of electrons into the conduction band of ZnMgO, resulting in 3 – 6 orders of magnitude improvement in the n‐type conductivity (Figure 9e). The study linked the positive aging effect to uncontrolled doping of ZnMgO by acid‐containing encapsulation resins.^[^ ^103^, ^104^, ^105^ ^]^ In contrast, the proposed reductive doping strategy achieved controlled n‐doping of ZnMgO (Figure 9f), eliminating the randomness and irreproducibility associated with resin‐induced positive aging. Shelf‐stable QD‐LEDs of all three primary colors were fabricated, where the blue‐emitting QD‐LEDs achieved an EQE of 21.1%, EL at 473 nm, and an exceptional peak brightness of 207000 cd m^−2^.^[^ ^103^ ^]^

## 5 Outlook and Future Directions

To conclude, we discuss several additional general directions that will be important to the development of blue QD‐LED technology to realize their full potential for next‐generation EL display and lighting applications.

### 5.1 Advances in Core/Shell Heterostructure Designs

ZnSeTe/ZnSe/ZnS QDs currently represent the frontrunning eco‐friendly candidates for blue emission, exhibiting device efficiency and operational stability rivalling those of state‐of‐the‐art Cd‐based blue QD‐LEDs. A key question moving forward is whether more advanced core/shell heterostructures can be developed beyond ZnSeTe/ZnSe/ZnS. One earlier report^[^ ^146^ ^]^ on blue QD‐LEDs used a ZnSeTe/ZnSe/ZnSeS/ZnS multi‐shell design with the additional ZnSeS intermediate shell, achieving 84% PLQY, narrow emission FWHM of 27 nm, and EQE of 9.5%, but no stability results were reported. The role of the ZnSeS intermediate shell is not fully understood, and this heterostructure has also received less attention. Separately, large ZnSe/ZnS nanocrystals (≈35 nm) have been synthesized with pure blue emission (455 – 470 nm), decent PLQY (60%), and narrow spectral linewidth (16 – 25 nm), but no device results were reported.^[^ ^45^ ^]^ Meanwhile, blue InP QDs are still underperforming due to persistent synthesis challenges, despite extensive research efforts.^[^ ^147^ ^]^ Developing synthetic methods such as high‐temperature molten salt annealing have produced bright red and green InGaP QDs^[^ ^148^ ^]^ as well as novel colloidal III‐V QDs^[^ ^149^ ^]^ and may provide another route to heavy metal‐free blue emitters in coming years.

### 5.2 HTL and HIL Advancements

Inefficient hole injection and instability of the organic hole transport and injection layers are general challenges for all QD‐LEDs, including red and green‐emitting devices. Developing novel materials or improving existing HTLs is necessary to optimize hole mobility, energy level alignment, and hole injection efficiency. Inorganic p‐type metal oxides are potential candidates due to their superior thermal and electrochemical stability compared to organic materials. However, their low conductivity and limitations in device performance and EQE must first be addressed. Meanwhile, relatively less attention has been dedicated to developing the HIL. PEDOT:PSS, originally adopted from OLED technology, currently remains the most widely used HIL for QD‐LEDs. While its sources of instability have been well‐studied for OLED devices,^[^ ^150^ ^]^ its specific contributions to QD‐LED degradation are less understood. Designing superior alternatives to PEDOT:PSS may be crucial for improving QD‐LED stability and efficiency.

### 5.3 Advanced Characterization and Mechanistic Studies

Studies of QD‐LED degradation have largely relied on a familiar set of characterization techniques, including electroabsorption, transient EL, capacitance‐voltage measurements, and impedance spectroscopy. While these methods have yielded valuable insights into red and green devices, they have not successfully pinpointed the unique mechanistic phenomena and degradation pathways in blue QD‐LEDs. Furthermore, single‐carrier device studies do not fully represent the working conditions of the complete QD‐LEDs since they overlook the critical roles of interfaces, band alignment, and charge transport, all of which have an outsized influence on the device EL characteristics. To address this gap, more mechanistic studies using advanced characterization tools on complete, representative device architectures are essential. For example, operando monitoring techniques would be valuable to reveal changes occurring in a QD‐LED during operation. The literature also lacks comparative studies that systematically examine the distinct mechanistic behaviors across blue, green, and red QD‐LEDs.

### 5.4 Stability Gap Between Spincoated and Patterned Blue QD‐LEDs

Among the various QD‐LED patterning techniques, inkjet printing has received the most attention due to its compatibility for large‐area, rapid, and cost‐effective deposition. While there is generally less literature on patterned QD‐LEDs, the reported operational lifetimes of blue QD‐LEDs typically decline sharply when transitioning from laboratory‐scale spincoating to scalable pixelation methods such as inkjet printing. Currently, the best‐reported T_50_ lifetimes^[^ ^151^, ^152^, ^153^ ^]^ for inkjet‐printed blue devices are on the order of 10^2^ h at 100 cd m^−2^. The underlying reasons for this large stability gap remain poorly understood. It is possible that the degradation mechanisms observed in studies of spincoated devices become aggravated in inkjet‐printed QD‐LEDs, but this hypothesis remains unproven. Any such study would also need to elucidate the physical processes responsible for the accelerated degradation. In contrast, inkjet printed red and green QD‐LEDs show much smaller stability losses, with published T_50_ reported lifetimes^[^ ^124^, ^151^, ^154^ ^]^ still exceeding 10^6^ h at 100 cd m^−2^. For comparison, blue‐emitting OLEDs found in pixelated displays exhibit T_50_ lifetimes^[^ ^30^ ^]^ of 10^6^ h under the same brightness conditions.

### 5.5 High Brightness Applications and Efficiency Roll‐Off

Most current research on blue QD‐LEDs focuses on optimizing device efficiency and stability at moderate brightness levels (<10^3^ cd m^−2^). While peak EQEs typically occur at a moderate current injection and brightness, the EQE can roll off dramatically at higher brightness levels due to Auger recombination and Joule heating.^[^ ^20^, ^155^ ^]^ This may limit the viability of blue QD‐LEDs for high brightness applications such as outdoor displays, laser diodes, and AR/VR systems. For example, brightness levels of >10^5^ cd m^−2^ are necessary for displays to be visible outdoors under intense sunlight. Red‐ and green‐emitting QD‐LEDs have demonstrated promising results at high operating brightness^[^ ^20^, ^156^ ^]^ by tailoring the QD structure to suppress Auger recombination and using a silicon substrate for effective heat dissipation. However, the challenge will be greater for blue QD‐LEDs, which have short lifetimes even at low operating brightness.

The recent few years have seen substantial progress in the development of blue QD‐LEDs. High‐performance, heavy metal‐free blue emitters, especially ZnSeTe/ZnSe/ZnS QDs, now enable QD‐LED devices with EQEs exceeding 25% and pure blue emissions close to 467 nm. Several challenges remain before practical usage and commercial application, particularly their limited operational lifetime. Progress in improving blue QD‐LED stability has been hindered by the lack of understanding regarding device failure mechanisms. However, the remarkable success of red and green devices offers optimism that with continued research, breakthroughs in developing blue QD‐LEDs are within reach.

## Conflict of Interest

The authors declare no conflict of interest.
